# Elucidating the spatio-temporal dynamics of the *Plasmodium falciparum* basal complex

**DOI:** 10.1371/journal.ppat.1012265

**Published:** 2024-06-03

**Authors:** Alexander A. Morano, Ilzat Ali, Jeffrey D. Dvorin

**Affiliations:** 1 Biological and Biomedical Sciences, Harvard Medical School, Boston, Massachusetts, United States of America; 2 Division of Infectious Diseases, Boston Children’s Hospital, Boston, Massachusetts, United States of America; 3 Department of Pediatrics, Harvard Medical School, Boston, Massachusetts, United States of America; University of Geneva Faculty of Medicine: Universite de Geneve Faculte de Medecine, SWITZERLAND

## Abstract

Asexual replication of *Plasmodium falciparum* occurs via schizogony, wherein 16–36 daughter cells are produced within the parasite during one semi-synchronized cytokinetic event. Schizogony requires a divergent contractile ring structure known as the basal complex. Our lab has previously identified PfMyoJ (PF3D7_1229800) and PfSLACR (PF3D7_0214700) as basal complex proteins recruited midway through segmentation. Using ultrastructure expansion microscopy, we localized both proteins to a novel basal complex subcompartment. While both colocalize with the basal complex protein PfCINCH upon recruitment, they form a separate, more basal subcompartment termed the posterior cup during contraction. We also show that PfSLACR is recruited to the basal complex prior to PfMyoJ, and that both proteins are removed unevenly as segmentation concludes. Using live-cell microscopy, we show that actin dynamics are dispensable for basal complex formation, expansion, and contraction. We then show that EF-hand containing *P*. *falciparum* Centrin 2 partially localizes to this posterior cup of the basal complex and that it is essential for growth and replication, with variable defects in basal complex contraction and synchrony. Finally, we demonstrate that free intracellular calcium is necessary but not sufficient for basal complex contraction in *P*. *falciparum*. Thus, we demonstrate dynamic spatial compartmentalization of the *Plasmodium falciparum* basal complex, identify an additional basal complex protein, and begin to elucidate the unique mechanism of contraction utilized by *P*. *falciparum*, opening the door for further exploration of Apicomplexan cellular division.

## Author summary

*Plasmodium falciparum* causes the most dangerous type of malaria, a disease that significantly impacts global health. One of the challenges in attempting to develop new antimalarial treatments is a lack of knowledge about the basic biology of the parasite. Cell division is especially unique in *Plasmodium*, and the organization and mechanism of its cytokinetic ring, known as the basal complex, are poorly understood. Here, we use novel microscopy techniques to characterize the spatial and temporal complexity of the basal complex. We find that the basal complex is composed of two subcompartments which become distinct over the course of cell division, complicating the temporal map of the basal complex by observing slight differences in recruitment and removal time, and we localize a newly-identified, essential basal complex protein, PfCen2, to the basal subcompartment. Finally, we show that neither actin dynamics nor myosin activity are required for basal complex contraction, but that intracellular calcium signaling is. Our study thus provides new insights into the organization and mechanism of *Plasmodium’s* cytokinetic ring and highlights a uniquely divergent, potentially exploitable aspect of *Plasmodium* cell biology.

## Introduction

*Plasmodium falciparum* is a member of a highly divergent clade of single-celled obligate parasites known as the Apicomplexa and is responsible for the most severe form of malaria, an ancient disease that still causes significant morbidity and mortality. While significant progress has been made towards the eradication of malaria, progress has stalled in recent years, due in part, to the emergence of resistance to frontline antimalarials [1,2]. A persistent hurdle impeding progress towards the goal of malaria eradication is the lack of knowledge about basic biological processes in the parasite, and indeed among Apicomplexa in general [3,4]. *P*. *falciparum* cytokinesis, for example, differs significantly from model organism cytokinesis. The various Apicomplexan genera have fundamentally divergent asexual replicative processes such that knowledge about one organism’s cytokinesis cannot be confidently applied to another [5,6].

*Plasmodium* cell division, during the asexual replicative cycle in human red blood cells, occurs via a process known as schizogony, wherein 16–36 daughter cells are generated from a single mother cell / mature “schizont”. During the final stage of schizogony, known as segmentation, the mother cell undergoes a round of semi-synchronous karyokinesis coupled with cytokinesis [7–9]. This process utilizes a specialized structure known as the basal complex (BC), a cytokinetic ring essential for cytokinesis and the proper deposition of the inner membrane complex (IMC). The IMC, in turn, is required to ensure the proper shape and structure of nascent merozoites [10]. The IMC is a double membrane structure, characteristic of the alveolates and present in all Apicomplexans, made of flattened vesicles and proteins underlying the plasma membrane [11–14] that ensures developing merozoites retain a rigid structure necessary to prevent deformation during invasion [15–17]. The IMC also serves as the anchor point for the actin-myosin motor that powers the invasion process after merozoite egress.

The basal complex (BC) is located at the leading edge of the IMC during segmentation and is predicted to be a contractile ring. It begins to be constructed in early segmentation, forming at the apical end of the nascent merozoite around 40 to 42 hours post invasion. As it is constructed, the BC’s diameter increases until about 46 HPI. During the final hours of segmentation, the basal complex contracts, and it resides at the basal ring of post-egress merozoites [6,10,18]. Compared to well-studied model organisms, the molecular mechanisms of the basal complex are poorly understood. The specialized processes/mechanisms of *P*. *falciparum* asexual replication require a cytokinetic ring with unique properties–for example, the formation of 16–36 daughter cells from a single shared cytoplasm demands cytokinetic rings that expand in synchrony, to roughly the same diameter, and simultaneously respond to cues to end the construction phase and begin contracting, whatever those may be [18]. In spite, or perhaps because of, the deep complexity required for proper *Pf* basal complex function, less than a dozen *Plasmodium* basal complex proteins have been identified, and only four (PfCINCH/PF3D7_0407800, PfMORN1/PF3D7_1031200, PfBLEB/PF3D7_0704300, and PfPPP8/PF3D7_1018200) have been extensively characterized with functional genetics [10,18–20]. Within this subgroup, only PfCINCH and PfPPP8 are strictly required for successful segmentation, though how each protein contributes to the structure and function of the basal complex is not well understood [10,18].

In recent years, detailed maps of basal complex protein recruitment and disassociation have been determined in the related Apicomplexan parasite, *Toxoplasma gondii*, including the identification of individual subcompartments [21–25]. As of 2022, four distinct subcompartments of the *T*. *gondii* basal complex have been described; the basal pole, the posterior cup, a third compartment apical to the former, and a fourth compartment more basal to the latter [22]. Proteins both early in recruitment and present only during / after BC contraction in *T*. *gondii* have been identified, and it has been shown that depletion of proteins like TgMyoJ impacts the localization of other proteins within the same subcompartment, perhaps impacting the very formation of said subcompartment [22]. Further, the *T*. *gondii* basal complex has been implicated in multiple functions beyond maintaining / performing constriction of daughter cells / IMC buildup during endodyogeny. In *T*. *gondii*, there is evidence that the BC is the site of IMC formation/construction (adding more membrane to IMC), as well as serving as the docking site to add cytoskeletal material. There is also evidence that implicates the *T*. *gondii* BC in apicoplast and mitochondrial segmentation, maintenance of the cytoplasmic bridge between daughter parasites, and possibly intravacuolar network (IVN) formation, nutrition acquisition, and motility [23]. Finally, a hypothetical “stretchy rubber band” mechanism of BC expansion/ contraction has been proposed to account for discrepancies between its seeming function as a contractile ring and the relative toleration of a complete TgMyoJ deletion, this being the primary myosin protein permanently localized to the basal complex [21,23]. In comparison, much less is known about how the basal complex might function in *P*. *falciparum*, and our understanding of how different BC proteins in *P*. *falciparum* relate to each other spatially and temporally is rudimentary at best.

While recent work from our lab has determined that the basal complex in *P*. *falciparum* exhibits temporal complexity, and that proteins are actively recruited and removed from the basal complex, the smaller size of this organism has hindered the discovery of spatial complexity in the *P*. *falciparum* basal complex, despite this being a known aspect of the *T*. *gondii* basal complex for over a decade [18,26,27]. In this paper, we report the discovery of two separate subcompartments in the *P*. *falciparum* basal complex, which we label the posterior cup and the main ring. Both homologs of known posterior cup basal complex proteins in *T*. *gondii* and *Plasmodium*-specific basal complex proteins are shown to localize to this compartment, speaking to the mix of conserved and divergent mechanisms defining the *P*. *falciparum* basal complex. We also demonstrate using ultrastructure expansion microscopy (U-ExM) and live-cell microscopy that these posterior-cup proteins are unevenly removed from the basal complex, likely into the residual body, at the end of segmentation. We further provide additional complexity to the initial temporal map of the *P*. *falciparum* basal complex by showing that PfSLACR/PF3D7_0214700 is recruited to the basal complex slightly earlier than PfMyoJ/PF3D7_1229800 using multiple dual-tagged live cell parasites strains.

We then show that PfMyoJ, despite having a similar temporal and spatial localization pattern to TgMyoJ, does not appear to play a similar functional role to its *T*. *gondii* counterpart. We then show that multiple nonessential *Plasmodium* basal complex proteins whose *T*. *gondii* homologs cause phenotypes can be knocked out without detectable consequences for *P*. *falciparum* in terms of growth, replication, or basal complex integrity and function. Unlike *T*. *gondii* [25], treating *P*. *falciparum* with an inhibitor of actin polymerization does not prevent basal complex contraction. However, we demonstrate that, as in *T*. *gondii*, PfCen2 localizes to the basal complex, along with a multiplicity of other cellular compartments [25,26,28]. We then generate an inducible knockdown of PfCen2, a protein whose endogenous locus has evaded modification for years, even in the *P*. *berghei* system [29–31]. Next, we determine that, among many defects, PfCen2-deficient parasites cannot fully contract their basal complexes, indicating that PfCen2, like TgCen2, is required for the final stage of basal complex constriction [25]. Finally, we demonstrate that treatment with a calcium chelator prevents the latter half of basal complex contraction, suggesting the utilization of calcium-responsive mechanisms for basal complex contraction, although failure to contract in response to calcium ionophore treatment indicates a degree of regulation preventing premature contraction beyond what is present in *Toxoplasma* [26]. Altogether, we demonstrate that some mechanisms of division used to construct and constrict the basal complex differ significantly between *T*. *gondii* and *P*. *falciparum* while others seem to be broadly conserved, if differently modulated [25,26].

## Results

I) *PfSLACR and PfMyoJ localize to a BC subcompartment basal to PfCINCH*

Previously, our lab identified PF3D7_0214700, named PfSLACR for small late-arriving contractile ring protein, and PfMyoJ as BC proteins recruited at the midpoint of segmentation, when construction and expansion of the BC is complete but contraction has not yet begun [18]. While PfSLACR is unique to *Plasmodiidae* (*Plasmodium* spp. and *Hemosporidia*), PfMyoJ has a homolog in *T*. *gondii*, TgMyoJ, which is also recruited to the expanded basal complex [24,25] and was one of the first proteins identified within a subcompartment of the *T*. *gondii* basal complex known as the posterior/basal cup [26]. Since PfMyoJ’s temporal localization reflects TgMyoJ’s, we wanted to determine whether its spatial localization does as well, which would demonstrate that the *P*. *falciparum* basal complex has spatial complexity. We examined PfSLACR’s spatial localization as well since it has a similar temporal localization to PfMyoJ.

**Fig 1:**
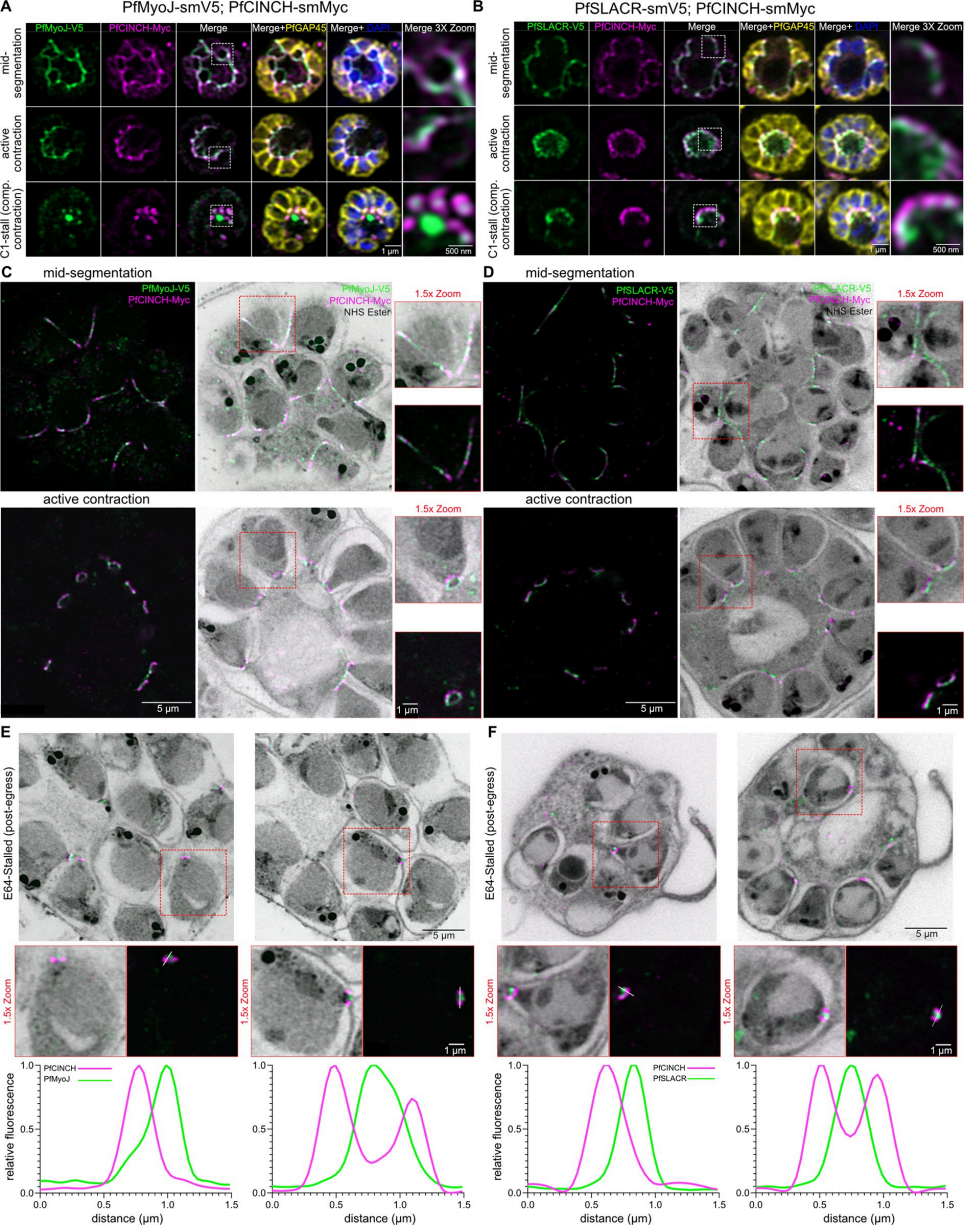
PfMyoJ and PFSLACR localize to a separate BC subcompartment basal to PfCINCH. **A)** Immunofluorescence of PfMyoJ and PfCINCH at mid-segmentation, active contraction, and completed contraction (Compound 1-stalled) with inner membrane complex marker PfGAP45. White boxes on merge panel indicate the 3x-zoomed region shown in the rightmost panel. **B)** Same as **A)** but with PfSLACR instead of PfMyoJ. **C)** U-ExM of PfMyoJ and PfCINCH during mid-segmentation and active contraction; images are of the same cell with (right) and without (left) NHS Ester (grey). Red boxes on the NHS Ester-merged image indicate the 1.5x-zoomed region to the right of each panel. **D)** Same as **C)** but with PfSLACR instead of PfMyoJ. **E)** Multiple U-ExM slices of E64-stalled schizont showing PfMyoJ and PfCINCH in the basal complex. Red boxes indicate the 1.5x-zoomed region below each slice. White lines drawn across the basal complex in the PfMyoJ and PfCINCH-only panels correspond to plotted fluorescence intensity traces below. **F)** Same as **E)** but with PfSLACR instead of PfMyoJ. Scale bar = 1 μm, except for the non-insets of **C)**-**F)**, where scale bar = 5 μm and the zoom panels of **A)** and **B)** where scale bar = 500 nm.

To compare the localization of these proteins to other basal complex proteins, we tagged PfCINCH, a highly abundant basal complex protein expressed throughout segmentation, with the spaghetti monster smMyc tag [32] in both the PfMyoJ-smV5 and PfSLACR-smV5 backgrounds, generating the lines PfMyoJ-smV5; PfCINCH-smMyc and PfSLACR-smV5; PfCINCH-smMyc. We visualized both transgenic strains by immunofluorescence at multiple stages of segmentation: mid-segmentation (when the basal complex is at its widest point), active/mid-contraction (when the basal complex is actively contracting) and completed contraction (post-segmentation, pre-egress) (Fig 1A and 1B). During mid-segmentation and active BC contraction stages, there was no discernible difference in localization between PfCINCH and PfMyoJ or between PfCINCH and PfSLACR (Fig 1A and 1B). To evaluate parasites that have completed basal complex contraction but have not ruptured their parasitophorous vacuolar membrane (PVM), we treated cultures with compound 1 (4-[2-(4-fluorophenyl)-5(1-methylpiperidine-4-yl)-1H-pyrrol-3-yl]pyridine or C1), a reversible protein kinase G inhibitor that prevents PVM rupture upon schizont maturation [33,34]. In C1-stalled (completed contraction) parasites, both PfMyoJ and PfSLACR appeared as distinct ‘dots’ in the center of and basal to the PfCINCH-smMyc ring (Fig 1A and 1B).

Quantifying this difference in localization was not possible with standard immunofluorescence due to resolution limits. Therefore, we utilized ultrastructure expansion microscopy (U-ExM) to determine with more confidence whether PfMyoJ and PfSLACR localized basal to PfCINCH near the end of segmentation and if so when this separation of compartments began [35,36]. U-ExM confirmed that during mid-segmentation (at the time of their recruitment) PfMyoJ and PfSLACR nearly colocalize with PfCINCH, with a very slight distance between each protein and PfCINCH (Fig 1C and 1D). U-ExM, with its increased spatial resolution, revealed that in late segmentation, while the basal complex is contracting, the distinct localizations of PfMyoJ and PfSLACR become more evident, and they begin to form distinct rings basal to the PfCINCH-containing ring (Fig 1C and 1D). In both lines, PfMyoJ and PfSLACR are consistently localized closer to the basal end of developing merozoites than PfCINCH regardless of the merozoite’s orientation. When contraction is complete in post-egress merozoites stalled with E64, PfMyoJ (Fig 1E) and PfSLACR (Fig 1F) appear as “dots” basal to PfCINCH’s ring and the ring that is visualized with the general protein stain, AlexaFluor405-N-hydroxysuccinimide ester (NHS Ester). E64 is a cysteine protease inhibitor that prevents RBC membrane degradation following rupture of the PVM, allowing for the visualization of merozoites that would have been released following egress (“post-egress”) [37,38]. Comparing fluorescence profiles of both proteins in E64-stalled parasites, PfMyoJ’s fluorescence peak is closer to the basal end of the parasite when an apical-basal line is drawn than PfCINCH’s, demonstrating its basal localization (Fig 1E, first inset). When a similar line is drawn through a cross-section of the basal complex, PfMyoJ forms a single peak of fluorescence as opposed to the dual peaks of PfCINCH (Fig 1E, second inset), corroborating its appearance as a single “dot” concentric to PfCINCH, and demonstrating its localization in *P*. *falciparum*’s “basal cup”, in contrast to the “main ring” defined by PfCINCH. PfSLACR, similarly, is found to localize both basal to (Fig 1F, first inset) and in between the peaks of (Fig 1F, second inset) PfCINCH. These two proteins initially define the basal cup in *P*. *falciparum*.

II) *PfMyoJ and PfSLACR only localize to this basal subcompartment in late segmentation*

Having identified specific subcompartment(s) within the basal complex, we sought to identify when this separation becomes apparent. To compare localizations of PfMyoJ and PfSLACR to PfCINCH in a robust and quantitative manner and because this change in localization happens entirely during the last two hours of schizogony, we selected four distinct “pseudo-timepoints” within this window: mid-segmentation (TP1), active contraction (TP2), completed contraction, pre-egress (C1-stalled) (TP3), and post-egress (E64-stalled) (TP4). Treating parasites with C1 or E64 are straightforward methods to distinguish between immediately pre-egress and post-egress phenotypes. We wanted to quantify the distance between PfCINCH and PfMyoJ or PfSLACR at each timepoint. Therefore, we captured five schizonts for each timepoint and selected eight basal complex rings per schizont (total 40 BC rings), oriented in either a three-quarters or full-on view, and for each ring drew three to four lines crossing through its center once and diameter twice using regularized guidelines detailed in the methods. We plotted the fluorescence intensity for both channels (PfSLACR/PfMyoJ and PfCINCH) along each line, such that two peaks for each color were generated corresponding to where each line crossed the BC diameter (Fig 1A and 1B). The distances between peaks of PfCINCH-smMyc and PfSLACR/PfMyoJ-smV5 were then measured (. Fig 1B and 1C).

**Fig 2:**
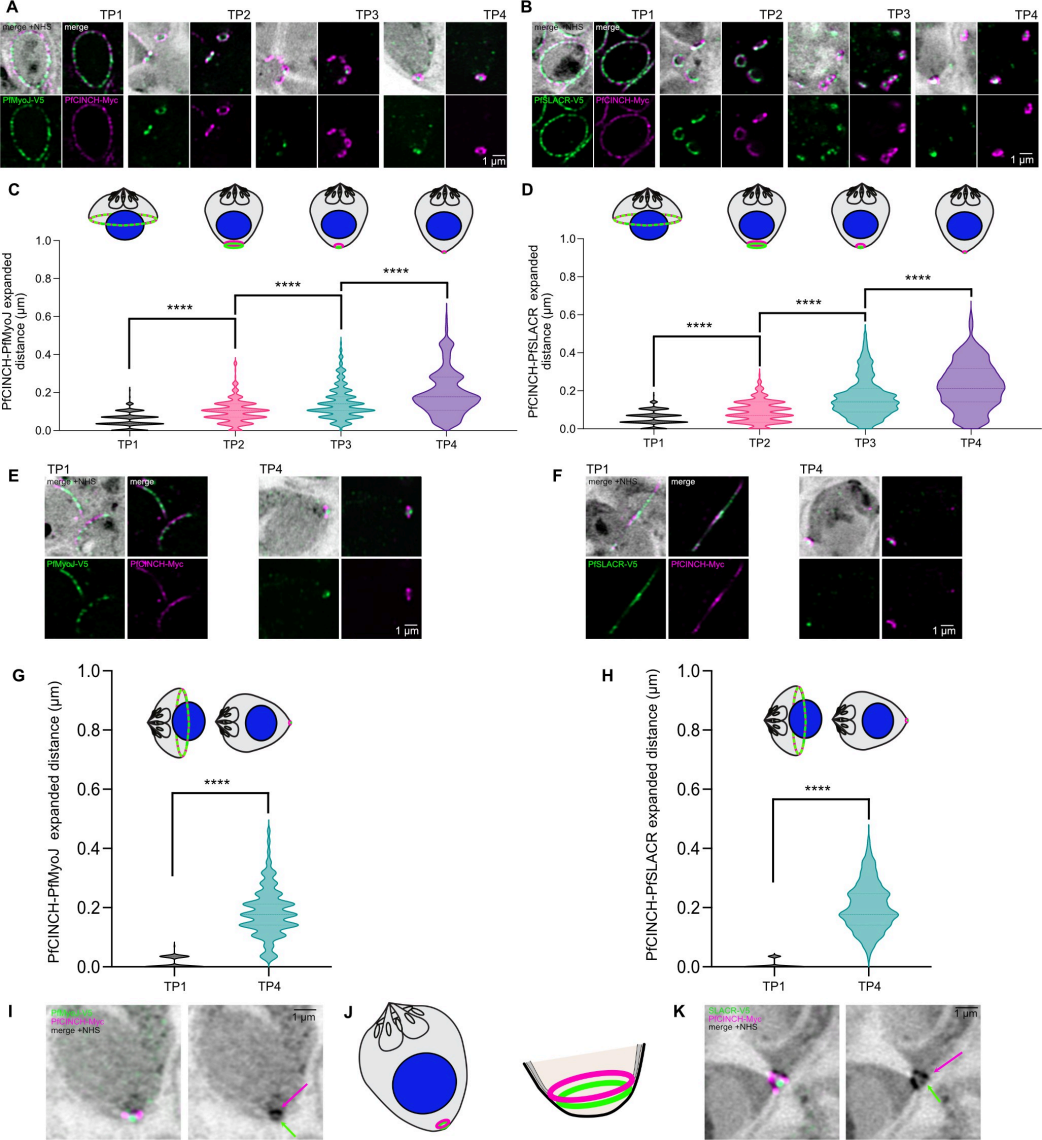
PfMyoJ and PfSLACR initially nearly colocalize with PfCINCH but localize to a more basal BC subcompartment in later segmentation. **A)** U-ExM localization of PfMyoJ and PfCINCH at four time points defined by BC appearance: mid-segmentation (TP1, ~46 HPI), active contraction (TP2, ~47 HPI), complete contraction (TP3, ~48 HPI), and post-egress (TP4, >48 HPI and E64-stalled). **B)** Same as **A)** but with PfSLACR instead of PfMyoJ. **C)** Quantification of expanded distance between PfMyoJ and PfCINCH fluorescence intensity peaks. n = 40 BC rings, 250–300 measurements/time point. Graphical representations of each time point are presented above the data. **D)** Same as **C)** but with PfSLACR instead of PfMyoJ. n = 40 BC rings, 250–300 measurements/time point. **E)** U-ExM localization of PfMyoJ and PfCINCH in sideways-oriented rings for TP1 and 4. **F)** Same as **D)** but with PfSLACR instead of PfMyoJ. **G)** Quantification of expanded distance between PfMyoJ and PfCINCH fluorescence intensity peaks on sideways-oriented rings. n = 50–60 BC rings, 110–125 measurements/time point. Graphical representations of TP1/4 are presented above the data. **H)** Same as **G)** but with PfSLACR instead of PfMyoJ. n = 50–60 BC rings, 110–125 measurements/time point. **I)** U-ExM localization of PfMyoJ and PfCINCH in an E64-stalled post-egress merozoite with NHS ester intensity beneath the main BC ring. **J)** Schematic depiction of BC subcompartments in mature merozoites. **K)** Same as **I)** but with PfSLACR and PfCINCH. Magenta arrow in NHS-only panels indicates PfCINCH ring, green arrow indicates dot-like density beneath main ring corresponding to PfSLACR **K)** or PfMyoJ **I)**. The data in **C)**, **D)**, **G)**, and **H)** were analyzed with an unpaired two-tailed student’s t-test and are displayed as violin plots. **** = p<0.0001. All scale bars = 1 μm.

With this extensive quantification, we found that the distance between PfCINCH and PfMyoJ increased significantly between each time point (p<0.0001 for all), indicating that PfMyoJ forms a smaller, more concentric ring distinct from the PfCINCH ring during late segmentation (Fig 2A and 2C). Initially (TP1), the mean expanded distance between PfCINCH and PfMyoJ was 0.05±0.04 μm, which increased to 0.10±0.06 (TP2), 0.15±0.08 (TP3), and finally 0.20±0.12 μm (TP4) (Fig 2C). The distance between PfCINCH and PfSLACR also increased significantly between each time point (p<0.0001 for all), thus PfSLACR also gradually localizes also to the basal subcompartment (Fig 2B and 2D). The mean distance between PfCINCH and PfSLACR was 0.06±0.04 μm at TP1, 0.09±0.06 at TP2, 0.16±0.10 by TP3, and 0.23±0.12 μm by TP4(Fig 2D). At TP3 and TP4, there were still instances of nearly overlapping PfMyoJ-PfCINCH/PfSLACR-PfCINCH fluorescent peaks (< = 0.00 to 0.03 μm between fluorescence peaks) since TP3/4 PfMyoJ/PfSLACR ‘dots’ sometimes localized to the BC perimeter. Thus, the range of measurements for TP3/4 is greater than for TP1/2 (Fig 2C–2D).

This analysis determined that PfSLACR and PfMyoJ begin forming distinct, concentric, smaller rings at TP2 (active contraction) and appear as basal cup “dots” in TP3/4. To prove that these concentric, smaller rings were more basal, we focused on the rings that had *not* been analyzed initially–those positioned on their sides. Measurements were only possible at two time points for this analysis, the first (TP1, pre-contraction) and the last (TP4, post-egress) because only these points consistently had merozoites angled such that the basal complex was perfectly “sideways” (S1D Fig) in orientation (Fig 2E–2H). For this analysis, two lines perpendicular to two regions of the BC ring were drawn, resulting in two linear measurements per ring (S1D, S1E Fig). Lines were always drawn such that x = 0 was oriented apical to the BC. Thus, a more basal protein would have a fluorescence peak further along the line (Figs 2G–2H; S1D–S1F). At TP1, the mean distance between PfCINCH and PfMyoJ was 0.01±0.02 μm. For PfCINCH-PfSLACR, the mean distance was 0.01±0.01 μm, emphasizing that PfCINCH, PfSLACR, and PfMyoJ begin segmentation in an overlapping subcompartment. However, at TP4 there was clear separation between PfCINCH and PfSLACR/PfMyoJ. The mean expanded distance between PfCINCH and PfMyoJ at TP4 was 0.18±0.08 μm, with a maximum distance of 0.46 μm, and between PfCINCH and PfSLACR at TP4, the mean distance was 0.20±0.08 μm, with a maximum distance of 0.42 μm (Fig 2G and 2H). PfSLACR and PfMyoJ were thus significantly more basal, with fluorescence peaks further from x = 0, than PfCINCH at TP4 (p<0.0001 for both T-tests comparing mean distance at TP1 and TP4). In E64-stalled parasites, there was often no overlap between fluorescence channels (Fig 2I–2K). Further, while in almost every timepoint, the basal complex appears as a single thick band of protein when visualized with the general protein stain (AF405 N-hydroxysuccinimide[NHS]-ester), at TP4, the basal cup is a dark “dot” of protein density below the main ring corresponding to the dot of PfMyoJ/PfSLACR (Fig 2I–2K).

To confirm that PfMyoJ and PfSLACR localize to the same basal subcompartment, we generated two additional transgenic parasite strains: PfSLACR-smV5; PfMyoJ-smMyc and PfMyoJ-smV5; PfSLACR-smMyc. By standard immunofluorescence, immediately pre-egress parasites (TP3) showed colocalization of PfSLACR and PfMyoJ (S2A Fig). Via U-ExM, both proteins colocalize in late contraction (TP2), post-contraction (TP3), and post-egress parasites (TP4) (S2B Fig). To determine if both proteins were continually associated with each other and to eliminate the possibility that structurally similar tags (smV5 and smMyc) were enabling continued colocalization in fixed schizonts, we generated the dual-fluorescently tagged line PfMyoJ-mNeonGreen; PfSLACR-mCherry, which also showed extensive colocalization in late schizonts up to and following egress in live parasites (S2C Fig).

III) *PfMyoJ and PfSLACR are removed unevenly from the basal complex*

**Fig 3:**
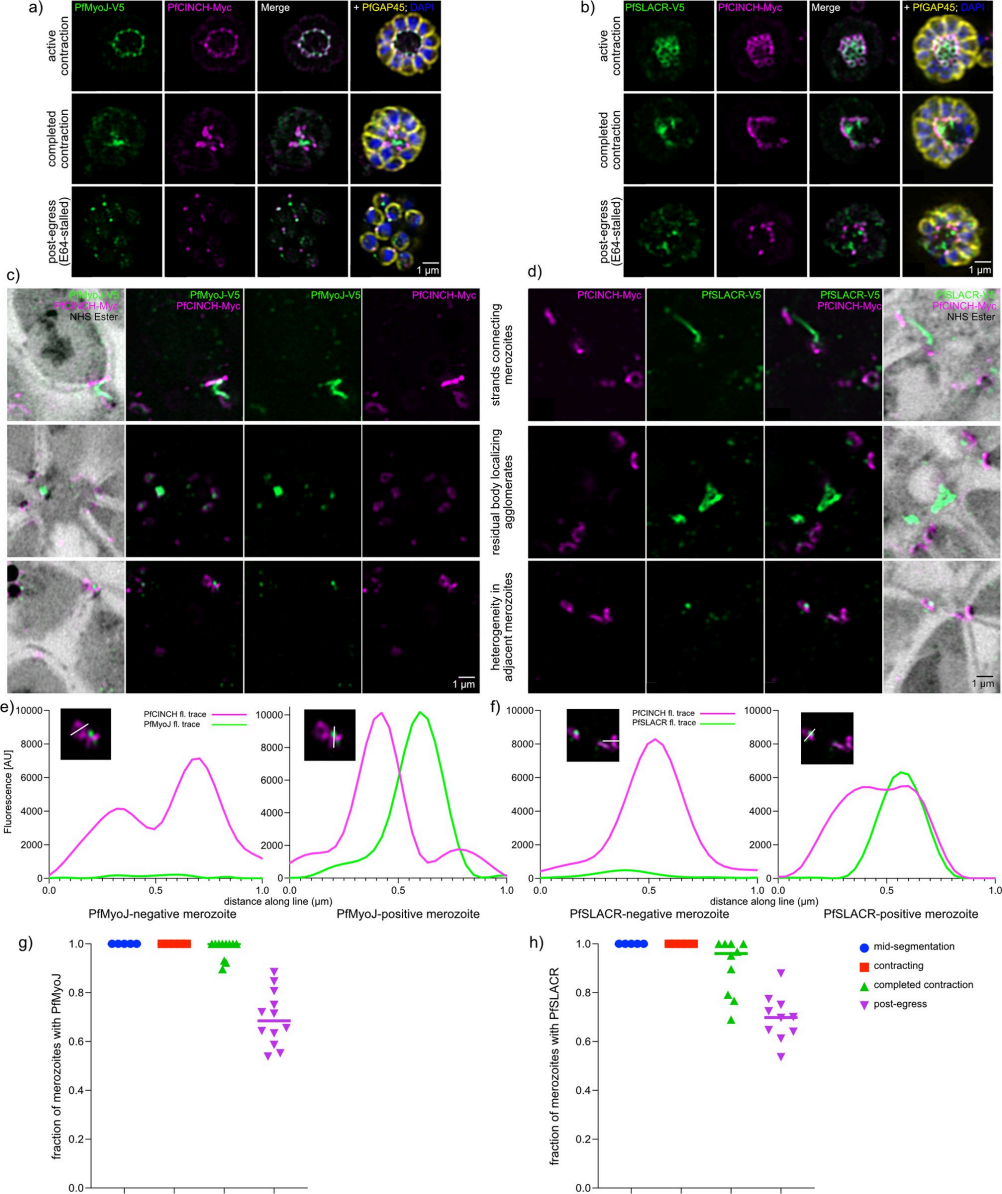
PfMyoJ and PfSLACR are partially removed from the basal complex at the end of segmentation. **A)** Immunofluorescence of PfMyoJ and PfCINCH during active contraction, completed contraction, and post-egress time points with inner membrane complex marker PfGAP45. **B)** Same as **A)** but with PfSLACR instead of PfMyoJ. **C)** U-ExM slices of late segmentation PfMyoJ-smV5; PfCINCH-smMyc parasites showing various non-basal complex PfMyoJ localization phenotypes. **D)** Same as **C)** but with PfSLACR instead of PfMyoJ. **E)** Graphs of fluorescence intensity for PfCINCH and PfMyoJ in adjacent merozoites, one PfMyoJ-negative (left) one PfMyoJ-positive (right). Images of BCs are in the top left of the graph with white lines indicating the fluorescence intensity trace. **F)** Same as **E)** but with PfSLACR instead of PfMyoJ. **G)** Quantification of PfMyoJ-positive merozoites in E64-stalled PfMyoJ-PfCINCH dual tagged parasites. n = 8–12 schizonts per time point, 25–35 merozoites per schizont. **H)** Same as **G)** but with PfSLACR-PfCINCH dual tagged parasites. n = 8–12 schizonts per time point, 25–35 merozoites per schizont. For **G)** and **H)**, individual values are shown along with the mean. All scale bars = 1 μm.

PfMyoJ and PfSLACR were uniformly bright at TP2 with little fluctuation between the BC rings within a single parasite (Fig 3A and 3B, top row). In immediately pre- and post- egress parasites (TP3 and TP4), some merozoites had far less PfSLACR or PfMyoJ, and agglomerates of PfSLACR/PfMyoJ were detected between merozoites (Fig 3A and 3B, bottom two rows). Some merozoites at TP4 had no detectable PfMyoJ/PfSLACR (Fig 3A and 3B, bottom row). In TP3 (post segmentation but pre-egress) parasites, heterogeneity of PfMyoJ/PfSLACR distribution between merozoites resulted in a diversity of phenotypes. Three of the most common are represented in Fig 3C: protein streaming out from the basal complex (Fig 3C and 3D, top row), protein agglomerates in what is likely the residual body (Fig 3C and 3D, middle row), and adjacent merozoites with disparate amounts of PfMyoJ/PfSLACR (Fig 3C and 3D, bottom row). PfSLACR or PfMyoJ-positive merozoites were often immediately adjacent to merozoites containing only PfCINCH. Quantitatively, fluorescence intensities of PfSLACR and PfMyoJ-positive merozoites were five to ten times higher in the PfSLACR/PfMyoJ channel than in adjacent merozoites that lacked evident PfSLACR/PfMyoJ, with minimal differences in PfCINCH fluorescence intensity (Fig 3E and 3F). PfMyoJ/PfSLACR-negative merozoites began to appear at TP3 and increased in frequency at TP4. In post-egress parasites, the mean frequency of PfMyoJ negative merozoites was 31±11% compared to 30±10% for PfSLACR-negative merozoites (Fig 3G and 3H). We evaluated synchronized PfMyoJ-smV5; PfSLACR-smMyc parasites to determine whether PfSLACR and PfMyoJ colocalized in the extra-basal complex protein streams and agglomerates. U-ExM on E64-stalled PfMyoJ-smV5; PfSLACR-smMyc parasites showed that PfSLACR and PfMyoJ were consistently colocalized in streams and agglomerates (S2D and S3C Figs). Further, merozoites which were negative for PfMyoJ were also negative for PfSLACR and vice versa (S2D Fig), with 80±14% of BCs being dual positive and 19±13% dual-negative. Only 1±2% and 0±1% were PfSLACR-positive only or PfMyoJ-positive only, respectively (S2E–S2F Fig). These results suggest that the two proteins are removed from the basal complex together, albeit not from every BC within a schizont (S2F Fig).

**Fig 4:**
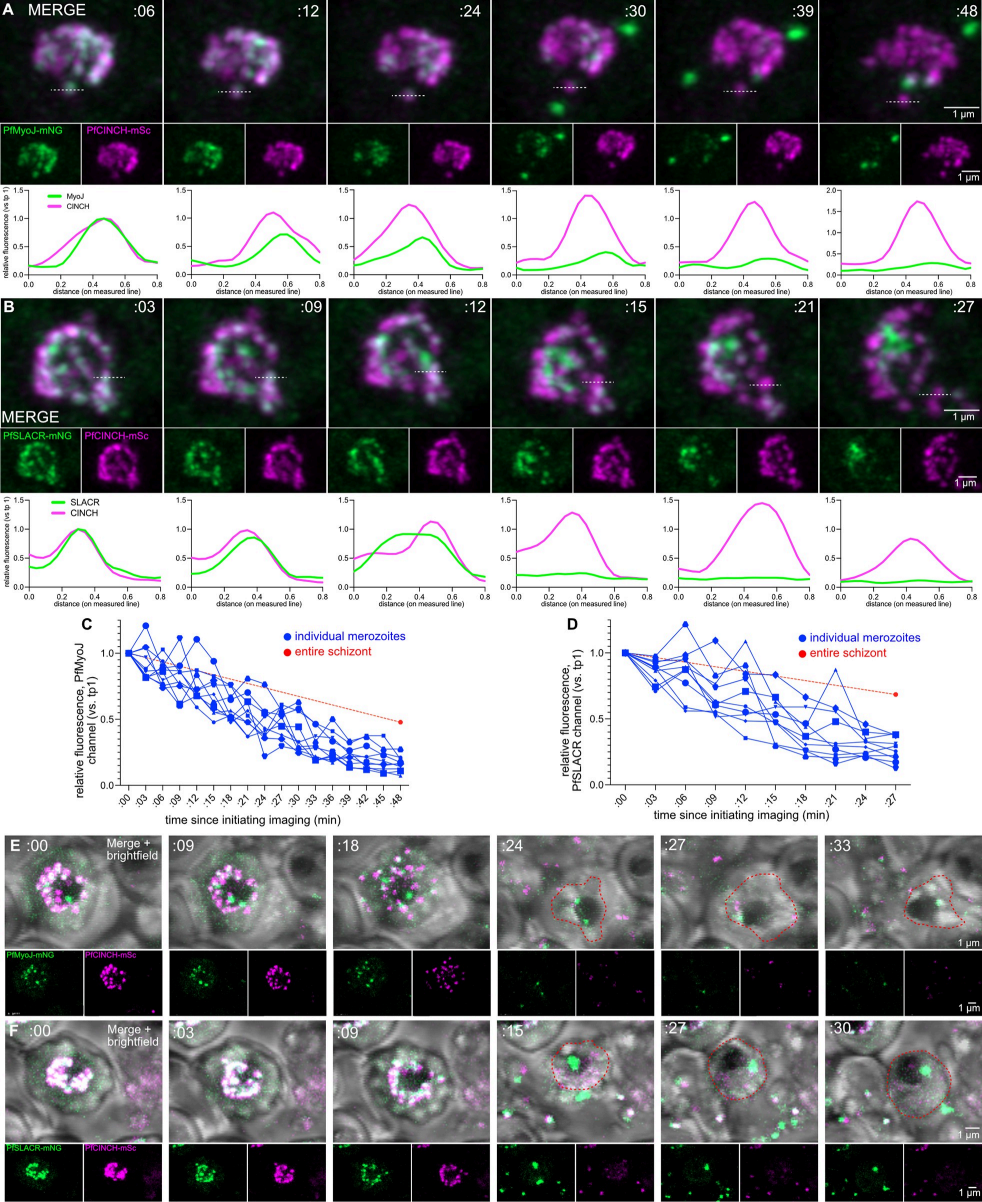
PfMyoJ and PfSLACR are removed from the basal complex to the residual body. **A)** Selected time points from Video 1 of live cell imaging of PfMyoJ-mNeonGreen (mNG); PfCINCH-mScarlet (mSc) parasites starting immediately pre-egress. Below images of each time point are graphs of fluorescence intensity for PfMyoJ and PfCINCH in a selected merozoite, with white lines indicating the fluorescence intensity trace and the maximum fluorescence at each time point for each channel normalized to the maximum fluorescence at the beginning of imaging. **B)** Same as **A)** but images are from Video 2 with PfSLACR-mNeonGreen. **C)** Plot comparing changes in relative fluorescence intensity of selected individual merozoites (in the PfMyoJ channel) of schizont represented in **A)** to changes in the entire schizont. **D)** Same as **C)**, but with PfSLACR; graph made from data taken from schizont in **B)**. **E)** Selected time points of live cell imaging of PfMyoJ-mNG; PfCINCH-mSc parasites starting immediately pre-egress. Red dotted line delineates boundary of residual body in post-egress parasite. **F)** Same but with PfSLACR instead of PfMyoJ. All time displayed as hours:minutes, all scale bars = 1 μm.

To examine PfMyoJ/PfSLACR depletion from the basal complex in live parasites, we generated a series of dual-tagged fluorescent strains: PfMyoJ-mNeonGreen; PfCINCH-mScarlet and PfSLACR-mNeonGreen; PfCINCH-mScarlet. At the stage of maximal BC diameter, PfMyoJ-mNeonGreen (Fig 4A, S1 Video) and PfSLACR-mNeonGreen (Fig 4B, S2 Video), were initially evenly distributed amongst PfCINCH-mScarlet merozoites. As schizogony progressed, both proteins began to be depleted from some merozoites and enriched in others. Depletion also corresponded with accumulation of PfSLACR and PfMyoJ in extra-BC agglomerates, most frequently in the center of the segmenting schizont, likely the residual body (Figs 4A and 4B, S3A–S3B). Comparison of PfMyoJ and PfSLACR fluorescence intensities at each time point demonstrate that PfMyoJ/PfSLACR depletion from individual merozoites did not correspond with PfCINCH depletion, as PfCINCH fluorescence intensity remained around or above 50% of fluorescence intensity at the initiation of imaging (Fig 4A and 4B). By quantifying the relative changes in fluorescence intensity in individual merozoites versus in entire schizonts at each timepoint, we observe that PfSLACR/PfMyoJ are depleted from individual merozoites but retained within the schizont (i.e., not degraded) (Figs 4C and 4D, S3A–S3D). Individual merozoites’ relative fluorescence consistently decreased over 50% compared to the initial measurement, whereas when the entire schizont was measured, including extra-merozoite protein agglomeration(s), relative fluorescence remained above 50% compared to the first time point (Fig 4C and 4D). To confirm that the agglomerate of PfSLACR or PfMyoJ protein removed from individual merozoites localized to the center of the parasite, likely the residual body, transmitted light was used to directly visualize this organelle. As expected, in both PfMyoJ-mNeonGreen (Fig 4E), and PfSLACR-mNeonGreen (Fig 4F) parasites, protein removed from merozoites localized to what is likely the residual body (Fig 4E and 4F). Using the PfMyoJ-mNeonGreen; PfSLACR-mCherry line, we observed that PfSLACR and PfMyoJ were depleted simultaneously and localized to the same agglomerates, reifying our U-ExM data (S3E–S3F Fig). Thus, PfMyoJ and PfSLACR are likely removed from the basal complex together, simultaneously, between the completion of contraction and the onset of egress and are moved to the center of the parasite, and likely the residual body.

IV) *PfMyoJ is recruited slightly after PfSLACR*

**Fig 5:**
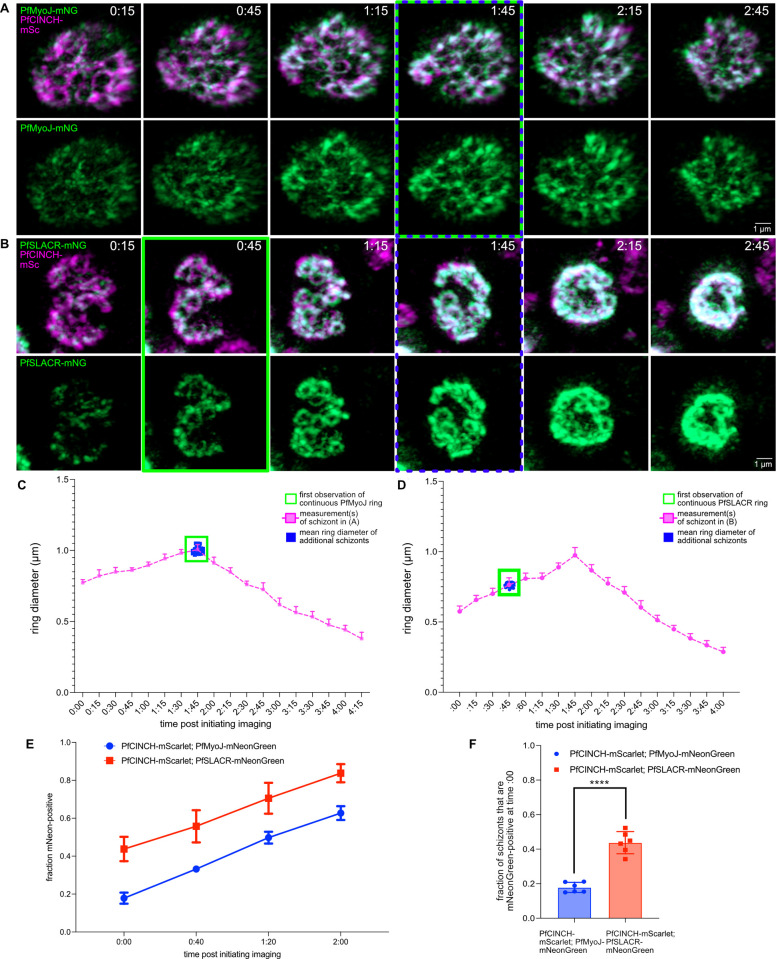
PfSLACR is recruited to the basal complex before PfMyoJ. **A)** Selected time points from Video 3 of live cell imaging of PfMyoJ-mNG; PfCINCH-mSc parasites. Green box = first observation of continuous PfMyoJ ring. Blue dashed box = BC diameter is greatest. **B)** Same as **A)** but with PfSLACR-mNG; PfCINCH-mSc parasites. Images from Video 4. **C)** Graph of mean BC diameter of PfMyoJ-mNG; PfCINCH-mSc parasites over time; pink line = measurements for schizont in **A)**. Green box = first observation of continuous PfMyoJ ring. Blue squares = mean BCDs of additional schizonts at the first observation of continuous PfMyoJ ring. **D)** Same as **C)** but with PfSLACR-mNG; PfCINCH-mSc parasites; pink line = measurements for schizont in **B)**. **E)** Graph comparing fraction of schizonts per field positive for PfSLACR (red) or PfMyoJ (blue) depending on strain. n = 6 fields/strain; 30–50 parasites/field. **F)** Graph comparing fraction of schizonts per field positive for PfSLACR (red) or PfMyoJ (blue) depending on line at the first time point (just after 44 HPI; t = 0:00). Time represented as hours:minutes. All scale bars = 1 μm. For **C)**, **D)**, **E)**, and **F)**, data are represented as mean ± SD. The data in **F)** were analyzed with an unpaired two-tailed student’s t-test and represented as mean ± SD. **** = p<0.0001.

During the short-interval time-lapse microscopy, PfSLACR appeared to form organized rings comparatively earlier than PfMyoJ. In PfMyoJ-mNeonGreen tagged parasites, there was detectable green fluorescence near the PfCINCH-mScarlet rings before the formation of an organized PfMyoJ ring. Furthermore, PfMyoJ slowly accumulated in punctate regions on the basal complex (Fig 5A, S3 Video). In comparison, in PfSLACR-mNeonGreen tagged parasites, there was no detectable green fluorescence prior to the punctate accumulation of PfSLACR-mNeonGreen along the BC ring–which were gradually filled in with PfSLACR (Fig 5B, S4 Video).

To quantify the timing of PfMyoJ or PfSLACR protein accumulation, the BC diameter (determined by PfCINCH-mScarlet) for each ring, at each time point, was measured, and the presence or absence of PfMyoJ-mNeonGreen or PfSLACR-mNeonGreen was noted in the respective strain. PfMyoJ /PfSLACR rings were considered present when the perimeter of each BC ring could be traced without disruptions in the PfMyoJ/PfSLACR channel, represented by the green-boxed time point in Fig 5C and 5D. Results for one schizont from each strain are represented by magenta lines in Fig 5C (PfMyoJ) and Fig 5D (PfSLACR); an additional replicate is depicted in S4A Fig (PfMyoJ) and S4B Fig (PfSLACR) with graphs in S4C Fig (PfMyoJ) and S4D Fig (PfSLACR). For 20–25 additional schizonts, we measured the BC diameter (BCD) for each ring at the first time point when PfSLACR or PfMyoJ were present. These data are represented as blue boxes in Fig 5C and 5D. This demonstrated that PfSLACR is recruited to the basal complex when the mean BCD is 0.76±0.20 μm, but PfMyoJ is not recruited until the mean BCD was 0.99±0.02 μm. (Fig 5C and 5D). When multiple schizonts’ entire timelines were plotted, mean BCDs were highly reproducible (S4E–S4F Fig).

Both lines (PfCINCH-mScarlet; PfMyoJ-mNeonGreen and PfCINCH-mScarlet; PfSLACR-mNeonGreen) were then simultaneously tightly synchronized to the same two-hour time window. Schizonts from both lines were then imaged simultaneously every 20 minutes, starting from when they were 44 to 46 hours post-infection (hpi). We then selected certain parasites per time point for analysis. We did not select parasites with basal complexes that were actively contracting, suggesting the schizonts were older than 46 hpi. We also did not select parasites with basal complexes that appeared as doublet rings, suggesting these schizonts were younger than 44 hpi. After this exclusion, 33 to 45 parasites per spatial position were selected to follow for eight consecutive time points. (Fig 5E and 5F). At T0:00, 18±3.0% of PfCINCH-mScarlet; PfMyoJ-mNeonGreen parasites were PfMyoJ-positive, while 44±6% of PfCINCH-mScarlet; PfSLACR-mNeonGreen parasites were PfSLACR-positive (Fig 5E and 5F). The fraction of PfSLACR-positive parasites increased to 56±9% after 40 minutes, 71±8% after 1 hour and 20 minutes, and 84±5% after 2 hours of imaging, compared to 33±2%, 50±3%, and 63±4% respectively in the PfCINCH-mScarlet; PfMyoJ-mNeonGreen line. For each time point, the % of mNeonGreen positive parasites in the PfCINCH-mScarlet; PfSLACR-mNeonGreen line was significantly greater (t = 0:00, p<0.0001 t = 0:40, p<0.0001; t = 1:20, p = 0.0002; t = 2:00, p<0.0001) than the % of mNeonGreen positive parasites in the PfCINCH-mScarlet; PfMyoJ-mNeonGreen line (Fig 5E). In a comparison of individual parasites matched by initial BCD, the PfSLACR-mNeon parasite became PfSLACR-positive 20 minutes after imaging began, whereas the PfMyoJ-mNeon parasite did not become PfMyoJ-positive until 1 hour after imaging began (S4G–S4H Fig).

V) *PfMyoJ and PfMORN1 are dispensable in combination for BC formation and function*

**Fig 6:**
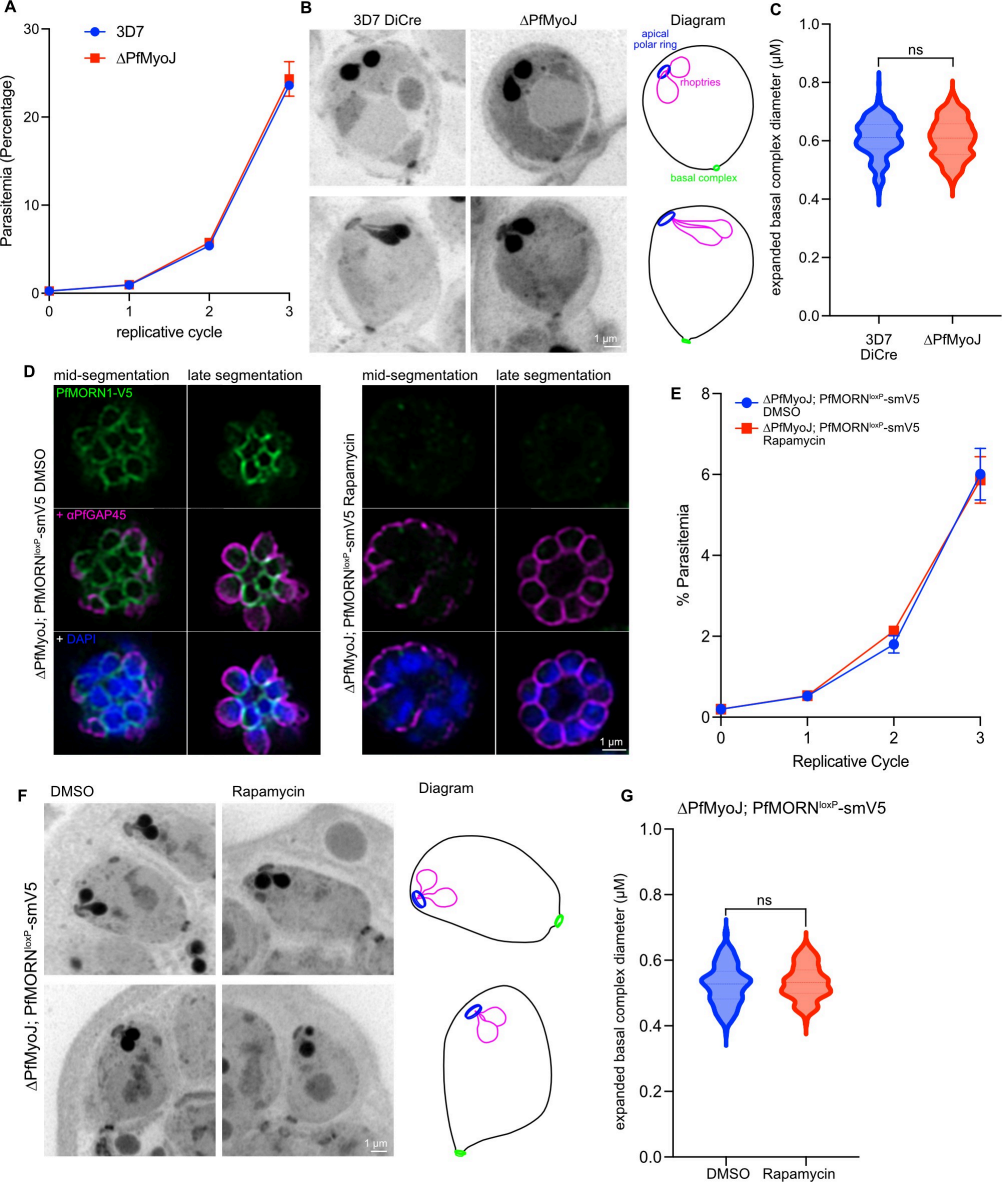
Knockout of PfMyoJ and/or PfMORN1 does not inhibit basal complex contraction. **A)** Flow cytometry-based replication curve comparing ΔPfMyoJ parasites to parental 3D7-DiCre line. **B)** Projections of 10–20 U-ExM slices comparing basal complex width in individual post-egress merozoites between ΔPfMyoJ and parental 3D7-DiCre parasites using AF405 NHS-Ester. A diagram illustrating basal complex orientation within the merozoite is adjacent to the ΔPfMyoJ images. **C)** Quantification of expanded basal complex diameter in ΔPfMyoJ and parental 3D7 parasites. n = 8–10 parasites, 240–260 basal complexes/condition. **D)** Immunofluorescence of PfMORN1-smV5 in ΔPfMyoJ background along with IMC marker PfGAP45 in DMSO (left) and rapamycin (right) treated parasites in mid- (left columns) and late (right columns) segmentation. **E)** Flow cytometry-based replication curve comparing replication rates of ΔPfMyoJ; PfMORN1^loxP^-smV5 parasites when treated with DMSO and rapamycin. **F)** Projections of 10–20 U-ExM slices comparing basal complex diameter in individual post-egress DMSO and rapamycin treated ΔPfMyoJ; PfMORN1^loxP^-smV5 merozoites using AF405 NHS-Ester. A diagram illustrating basal complex orientation within the merozoite is adjacent to the rapamycin images. **G)** Quantification of expanded basal complex diameter in DMSO and rapamycin treated ΔPfMyoJ; PfMORN1^loxP^-smV5 merozoites. n = 5–7 parasites, 130–160 basal complexes/condition. The data in **C)** and **G)** were analyzed with an unpaired one-tailed student’s t-test and are displayed as violin plots. ns = p>0.05. All scale bars = 1 μm.

PfMyoJ has a similar temporal and spatial localization to that observed for TgMyoJ in *T*. *gondii*. Furthermore, in *T*. *gondii* parasites with TgMyoJ deletion, the parasites had BC contraction deficiencies [24,25]. To evaluate if there are similar phenotypes in *P*. *falciparum*, we generated a PfMyoJ knockout strain (ΔPfMyoJ) using CRISPR-Cas9 and a double crossover mediated homologous recombination event that generated a 217 base pair deletion and inserted the hDHFR drug cassette in the middle of the gene sequence, which includes a transcription terminator, thus preventing production of PfMyoJ beyond the first 134 amino acids (out of 2,271 total amino acids) (S5A, S5C Fig). Unlike its *T*. *gondii* homolog, this transgenic parasite line lacked detectable qualitative or quantitative growth defects, although this is not surprising because PfMyoJ is predicted to be dispensable and PbMyoJ has been knocked out with no phenotypic consequences [39–41] (Figs 6A, S5B). The disruption of PfMyoJ was verified by whole-genome sequencing (sequence reads deposited in NCBI Sequence Read Archive, SRR19383288). To compare BC contraction between the ΔPfMyoJ and the parental 3D7-DiCre [42] parasite strains, the diameter of the BC for each merozoite was measured in E64-stalled schizonts by U-ExM (Fig 6B). Following 200 measurements, the mean expanded BCD of 3D7-DiCre parasites was 0.61±0.07 μm, compared to 0.61±0.06 μm for ΔPfMyoJ parasites (Fig 6C). The lack of knockout phenotype in *P*. *falciparum* compared to *T*. *gondii* is similar to the lack of phenotype observed for deletion of PfMORN1 [19,27,43,44]. To evaluate if a double knockout would have a detectable phenotype, we generated ΔPfMyoJ; PfMORN1^loxP^-smV5 parasites (S5D Fig). These ΔPfMyoJ parasites express the split Cre recombinase [42,45] that, upon addition of rapamycin, will dimerize and excise the coding region for PfMORN1. These parasites grew normally and PfMORN1 localized to the BC, as expected, in the absence of PfMyoJ (Fig 6D, left panel). Upon addition of rapamycin, PfMORN1 was deleted (S5D Fig), but segmentation, indicated by normal localization of the IMC-associated protein, PfGAP45 [46], and uniformly-sized merozoites, was not perturbed (Fig 6D, right panel). Flow cytometry-based replication assays showed no difference in replication rate between rapamycin or DMSO-treated ΔPfMyoJ; PfMORN1^loxP^-smV5 parasites (Fig 6E), and double-knockout clones were readily obtained (S5D Fig). The mean expanded BCD of ΔPfMyoJ; PfMORN1^loxP^-smV5 parasites treated with DMSO was 0.53±0.06 μm, compared to 0.53±0.05 μm for rapamycin-treated parasites (Fig 6F and 6G). Thus, PfMORN1 and PfMyoJ can be simultaneously knocked out without detectable phenotype during the asexual stage.

VI) *Basal Complex Contraction does not require actin dynamics*

The lack of a phenotype in the ΔPfMyoJ BC led us to question if actinomyosin interactions were important for BC contraction, potentially mediated by an unidentified myosin protein. Therefore, we next attempted to perturb basal complex contraction by disrupting actin filaments with cytochalasin D (cytoD). In *T*. *gondii*, 1 μM cytoD treatment for 4 hours before imaging inhibits complete contraction of the basal complex, resembling ΔTgMyoJ parasites [24,25]. To determine if this was true for *P*. *falciparum* parasites, we tested different concentrations of cytoD in a PfCINCH-mNeonGreen strain. Treatment with 1 or 2 μM cytoD from 30 minutes prior to and during imaging did not inhibit BC contraction or even BC formation: BCs of parasites which started imaging early during segmentation were able to fully contract before egress (S6A Fig). We counted the number of schizonts per field that progressed to egress during imaging and of these determined how many had BCs which appeared as fully contracted dots immediately pre-egress. 98±2.8% of DMSO-treated, 98±2.9% of 1 μM cytoD-treated, and 96±3.5% of 2 μM cytoD-treated parasites which egressed had fully contracted BCs, thus there was virtually no impact on BC contraction of cytochalasin D treatment (S6D Fig). Importantly, treatment with cytochalasin D did inhibit reinvasion of egressed parasites and proper segregation of the apicoplast, demonstrating that the compound was working as expected (S7A–S7C Fig) [47,48].

To verify that segmentation was progressing normally and to directly visualize IMC formation, we fused mCherry to PfIMC1c, an essential alveolin protein [15]. The resulting parasites allowed simultaneous visualization of the BC (PfCINCH-mNeonGreen) and the IMC (PfIMC1c-mCherry). We initially treated these dual-tagged parasites with 2 μM cytoD (or DMSO vehicle) immediately pre-imaging, which did not inhibit BC contraction: 98±2% of DMSO-treated and 97±2% of cytoD-treated parasites which egressed were able to fully contract their BCs (S6B, S6E Fig).

**Fig 7:**
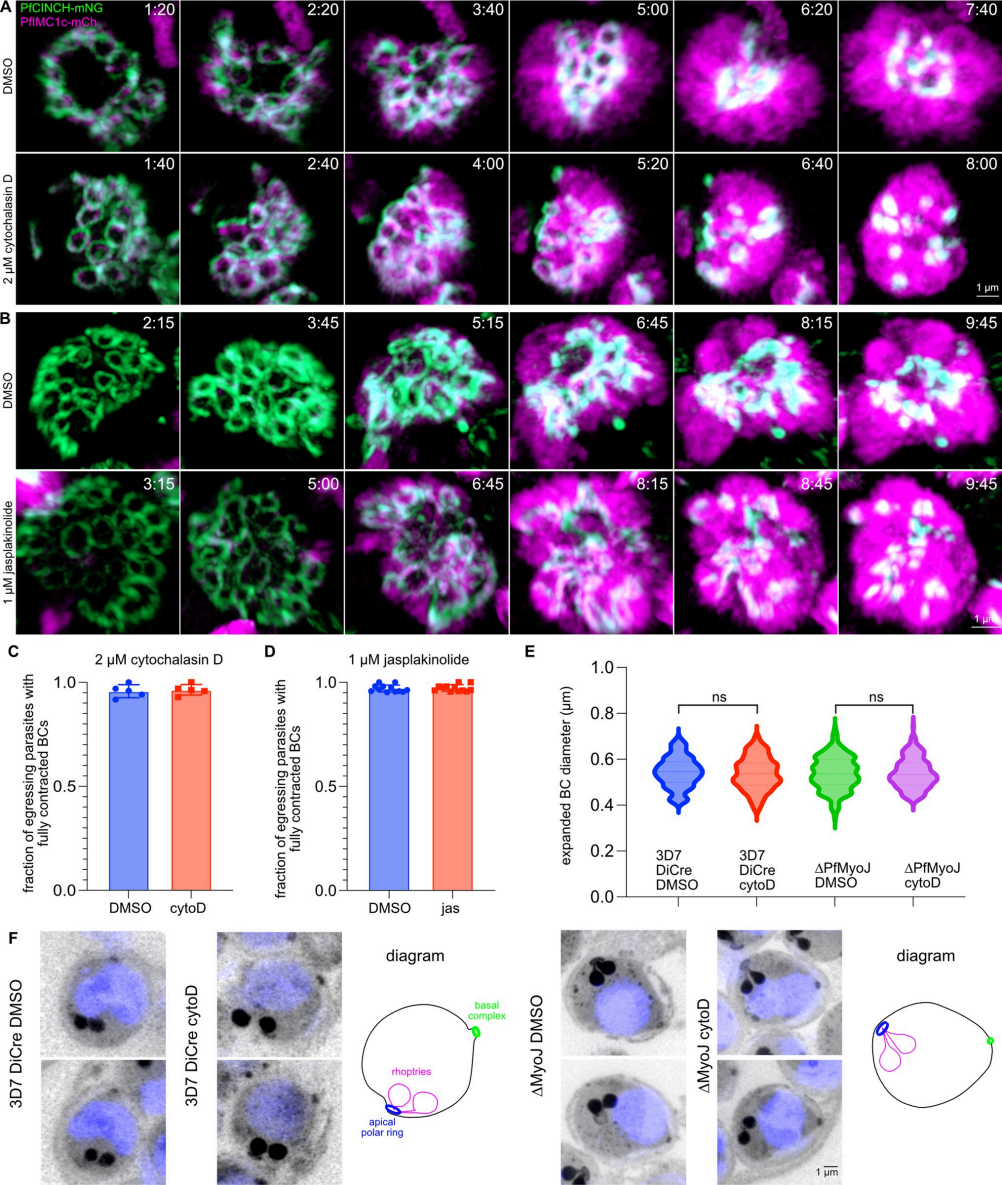
Chemical disruption of actin dynamics does not impact basal complex contraction. **A)** Selected time points of live cell imaging of PfCINCH-mNG; PfIMC1c-mCherry (mCh) parasites treated with DMSO (from Video 5, top row) or 2 μM cytoD (from Video 6, bottom row). **B)** Same as **A)**, but with 1 μM jasplakinolide (DMSO images from Video 7, jas images from Video 8). **C)** Fraction of egressing parasites per field with fully contracted basal complexes (BCs that contracted to the point that their diameter could not be measured) between DMSO and 2 μM cytoD-treated parasites. n = 6 fields/condition, 30–50 parasites/field. **D)** Fraction of egressing parasites per field with fully contracted basal complexes between DMSO and 1 μM jasplakinolide-treated parasites. n = 12 fields/condition, 40–60 parasites/field. **E)** Quantification of expanded basal complex diameter between 3D7-DiCre and ΔPfMyoJ DMSO/cytoD-treated parasites. n = 6–8 parasites; 150–250 measurements/condition. **F)** U-ExM slices of post-egress (E64 stalled) 3D7-DiCre and ΔPfMyoJ parasites treated with DMSO or 2 μM cytoD at 44 HPI and E64 at 46 HPI. A diagram illustrating basal complex orientation within the merozoite is adjacent to the U-ExM images. Time depicted as hours:minutes. The data in **C)** and **D)** are displayed as mean ± SD with individual values overlayed. The data in **E)** were analyzed with separate unpaired one-tailed student’s t-tests and are displayed as violin plots. ns = p>0.05. All scale bars = 1 μm.

Next, we pre-treated parasites with cytoD, as had been done in *T*. *gondii* [24,25]. 2 μM cytoD (or DMSO vehicle) was added 4 hours before schizonts were harvested for imaging. Under these conditions, basal complexes of cytoD-treated parasites were still able to expand, reach maximal diameter, and contract (Fig 7A, S5–S6 Videos). Again, even parasites that began imaging during early segmentation constructed BCs that fully contracted. For both DMSO and cytoD treated parasites, 96±3% of parasites which egressed had fully contracted BCs. (Fig 7C). To determine if increasing the concentration of cytoD would inhibit contraction, schizonts were treated with 4 μM cytoD/DMSO (S6C Fig). Even at this concentration of cytoD, 97±2% of parasites which egressed in both conditions could construct and fully contract the BC (S6C, S6F Fig).

To detect subtle differences not resolvable in our live cell imaging, U-ExM was utilized to allow higher resolution measurements of the BC diameter in wild type or transgenic parasites that had been treated with cytoD. Synchronized 44 hpi 3D7-DiCre [42] and ΔPfMyoJ parasites were treated with cytoD and E64 for 6 hours (Fig 7E and 7F) prior to harvest for U-ExM. There was no significant increase in BCD in cytoD-treated parasites compared to DMSO-treated parasites: mean (expanded) BCD in 3D7-DiCre DMSO-treated parasites was 0.55±.07 μm, for 3D7-DiCre cytoD-treated parasites, 0.54±0.8 μm, for ΔPfMyoJ DMSO-treated parasites, 0.54±.08 μm, and for ΔPfMyoJ cytoD-treated parasites, 0.54±.07 μm (Fig 7F). Thus, unlike in *T*. *gondii*, neither the specific action of PfMyoJ nor the maintenance of polymerized actin filaments contribute to basal complex contraction in *P*. *falciparum*.

We then wanted to determine whether BC contraction was inhibited when actin was not able to depolymerize within the parasite. Based on the literature and initial tests of our compound, we determined that 1 μM jasplakinolide (jas) was enough to inhibit depolymerization of actin and to prevent reinvasion of egressed parasites [49–51]. We added 1 μM jas or an equal volume of DMSO to imaging media both immediately and 2 hours before initiating imaging, and, in both experimental conditions, there was no significant increase in BCD for jas-treated parasites compared to DMSO-treated parasites; 97±1.7% of DMSO-treated and 97±1.6% of 1 μM-jas treated parasites which egressed fully contracted their BCs (Fig 7B, 7D, S7–S8 Videos). Even parasites which started imaging at the earliest stages of BC ring formation were able to instigate the construction of, expand, and contract the BC successfully while actin polymers were prevented from depolymerizing. Thus, the dynamic actin filaments which are essential to the contraction of model eukaryotic cytokinetic rings are entirely dispensable for cytokinesis in *P*. *falciparum* [52].

In *Toxoplasma*, TgMyoJ is only one protein known to participate in BC contraction. TgCen2, one of three centrins in the organism, localizes to the centrioles but also the apical annuli, the preconoidal ring at the apical tip of the parasite, and, notably, the basal complex [25,26,28]. Although TgCen2 has multiple localizations and thus likely multiple roles in the cell, the most obvious defect in *T*. *gondii* tachyzoite when TgCen2 is depleted using an inducible Tet-OFF system, is a failure of BC contraction similar to ΔTgMyoJ parasites [25,53]. This is likely because the Tet-OFF system does not fully repress transcription; indeed, TgCen2 remains present at the centrioles at least as faint dots in the presence of ATc, and, in a recent study utilizing a stronger inducible knockout system combining multiple technologies, TgCen2-deficient tachyzoites had more severe phenotypic consequences [25,28,53].

In our quest to identify proteins responsible for BC contraction, we thus wondered if PfCen2 had a similar localization and function in *P*. *falciparum*. We first attempted to endogenously tag PfCen2, utilizing small tags connected by flexible linkers, but no transfections endogenously tagging this protein with an epitope tag were successful, which was has been noted in previous work studying PfCen2 [29]. However, the small size of PfCen2 allowed us to generate multiple transgenic lines wherein the expression of a codon-altered copy of PfCen2 with an N-terminal epitope tag is driven by its native promoter from a non-integrated plasmid. Episomal expression of PfCen2 has previously been utilized, but this is the first time the protein has been expressed episomally under the control of its native promoter [29]. Previous work utilized the promoter of the chloroquine resistance transporter, PfCrt, which has significantly different transcriptional dynamics: PfCrt transcription is highest at 10 HPI and lowest at 40 HPI whereas PfCen2 transcription is highest at 40 HPI and lowest between 10–20 HPI [29,54].

**Fig 8:**
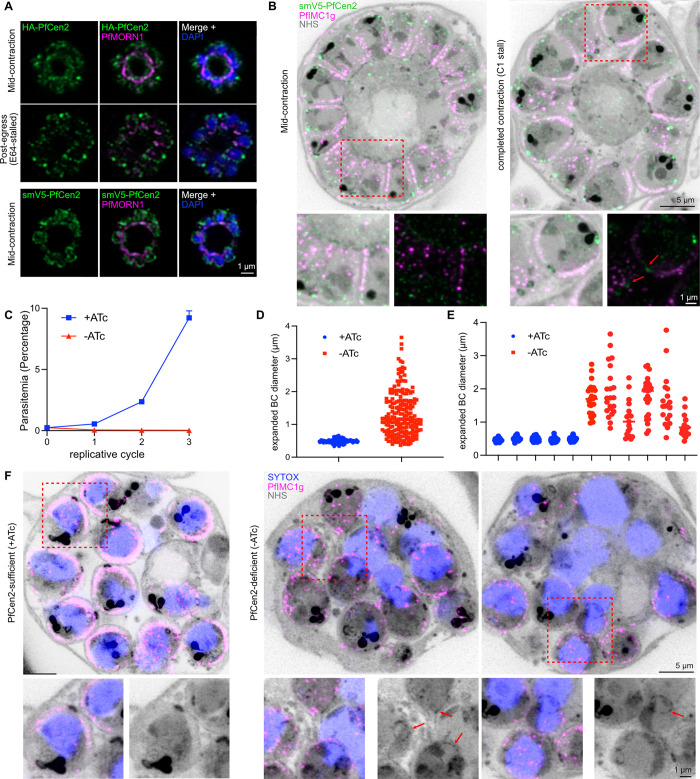
PfCen2 localizes to the basal complex and is essential for completing BC contraction. **A)** Immunofluorescence of episomally-expressed 2HA-PfCen2 and smV5-PfCen2 in mid-segmentation and post-egress (E64-stalled) parasites, with basal complex marker PfMORN1. **B)** U-ExM of episomally-expressed smV5-Cen2 and IMC-associated alveolin protein PfIMC1g in mid-segmentation and completed contraction (C1-stalled) parasites. Red boxes indicate the 1.5x-zoomed region below each panel; red arrows point to basal cup localization of smV5-PfCen2. **C)** Flow cytometry-based replication curve comparing PfCen2^Tet^ parasites in the presence (PfCen2-sufficient) and absence (PfCen2-deficient) of ATc. **D)** Quantification of expanded basal complex diameter between E64-stalled PfCen2-sufficient and -deficient PfCen2^Tet^ parasites. n = 6–10 parasites; 180–210 measurements/condition. **E)** Comparison of expanded basal complex diameters measured in 5 schizonts, demonstrating significant variance in PfCen2-deficient BCDs. **F)** U-ExM slices of post-egress (E64 stalled) PfCen2-sufficient and -deficient PfCen2^Tet^ parasites. Adjacent images in PfCen2-deficient condition show different slices of the same schizont. Red boxes indicate the 1.5x-zoomed region below each panel; red arrows point to enlarged basal complexes in two different areas of the schizont. All scale bars = 1 μm except the non-zoomed U-ExM panels in **F)** where scale bar = 5 μm.

We generated two lines containing episomally-expressed PfCen2; 2HA-Cen2 (Ep) and smV5-Cen2 (Ep) and saw that in early schizogony, both episomal Cen2 constructs localized around the dividing nuclei via immunofluorescence, aligning with previously published episomal expressions of PfCen2 (. S8A–S8B Fig) [29]. U-ExM further validated the efficacy of our episomal constructs: smV5-PfCen2 localized at the branches of the outer centriolar plaque, with the number of smV5-PfCen2 foci corresponding to the number of outer centriolar plaque branches, matching previous examinations of centrin localization via U-ExM (. S8B Fig) [55]. In later schizonts, PfCen2 adopted multiple localizations, like its *T*. *gondii* ortholog. Via traditional immunofluorescence, there was a strong signal at the apical end of the parasite likely corresponding to the apical polar ring, referred to as the preconoidal ring in *T*. *gondii* (a hypothesis verified with U-ExM) (Fig 8A and 8B) [26,28]. There was also staining near the apical end of the parasite but not directly correlated with the apical polar ring, potentially representing the apical annuli, to which TgCen2 has been localized in *T*. *gondii* and which have recently been discovered in *P*. *falciparum* [26,28,56]. There was also some remaining signal near the nuclei, but most importantly, colocalization with PfMORN1 at the basal complex in both the 2HA and smV5-Cen2 (Ep) lines (Fig 8A). Though the signal at the basal end of the parasite was not as strong as the apical end signal(s), as is true in *T*. *gondii*, 2HA-PfCen2 and smV5-PfCen2 still formed visible rings in actively contracting parasites (Fig 8A) [26,28]. Notably, even by traditional immunofluorescence, 2HA-PfCen2 seemed to localize to the center of the PfMORN1-defined basal complex in E64-stalled parasites (Figs 8A, S8A).

However, because *P*. *falciparum* is a smaller parasite, the multiple localizations of PfCen2 are not as appreciable as in *T*. *gondii* via traditional immunofluorescence [26,28]. Thus, we performed U-ExM on the smV5-Cen2 (Ep) line. In actively dividing schizonts, although there was a strong signal at the apical polar ring and the (putative) apical annuli, there was a fainter but still appreciable signal at the basal complex, despite the higher level of background. This dim staining is similar to what was observed for the basal complex staining of TgCen2 in immature tachzyoites undergoing endodyogeny (Fig 8B) [26,28]. In Compound 1-stalled schizonts, localization of PfCen2 to not just the basal complex but the basal cup in particular was more appreciable—here, the three strongest signals are the apical polar ring signal, the bright apically-oriented dot that could correspond to the apical annuli, and the basal complex, or rather, basal cup signal (Fig 8B). Because TgCen2 basal complex signaling is strongest in extracellular tachyzoites, we also looked at released merozoites in E64-stalled parasites and again saw a distinct signal more basal to the basal complex density (marked with NHS Ester) (S8C Fig) [26,28].

PfCen2 was predicted to be essential in a *P*. *falciparum* genetic screen [39]. Because of this, and our multiple failed attempts to endogenously tag this protein, we designed a construct that allowed for Tet-responsive translational control of PfCen2, modifying only the RNA transcript and not the produced protein. We made use of the TetR-DOZI system, appending an array of 10x TetR-binding RNA aptamers after the end of the PfCen2 ORF followed by the TetR-DOZI fusion protein expression cassette [10,18,57–59]. This strategy allowed us to modify the endogenous PfCen2 locus (S8D Fig). As expected, the protein was highly essential; removal of ATc led to a complete failure of replication (Figs Figs 8C and S8E). Examination of E64-stalled PfCen2-deficient parasites displayed significant segmentation defects; most relevantly, PfCen2-deficient merozoites had significantly larger basal complexes on average (p<0.0001). The mean expanded BCD in PfCen2-sufficient parasites was 0.49±0.70 μm, compared to 1.31±0.67 μm in PfCen2-deficient parasites (Fig 8D–8F). Both merozoites that had separated from the residual body and those that remained attached despite going through egress had larger basal complexes than PfCen2-sufficient parasites collected after egress (Fig 8F). However, this failure to fully contract was not consistent between schizonts or between merozoites within a schizont: some PfCen2-deficient merozoites had regularly-sized basal complexes and BCD variance within individual schizonts was significantly greater in the PfCen2-deficient condition (t-test comparing mean BCD p<0.0001; F test comparing variances in BCD p<0.0001) (Figs 8E, S8E–S8F). These data suggest that while PfCen2 is involved in BC contraction, it is not solely responsible, and that PfCen2 may have a role in mediating synchrony of BC contraction.

**Fig 9:**
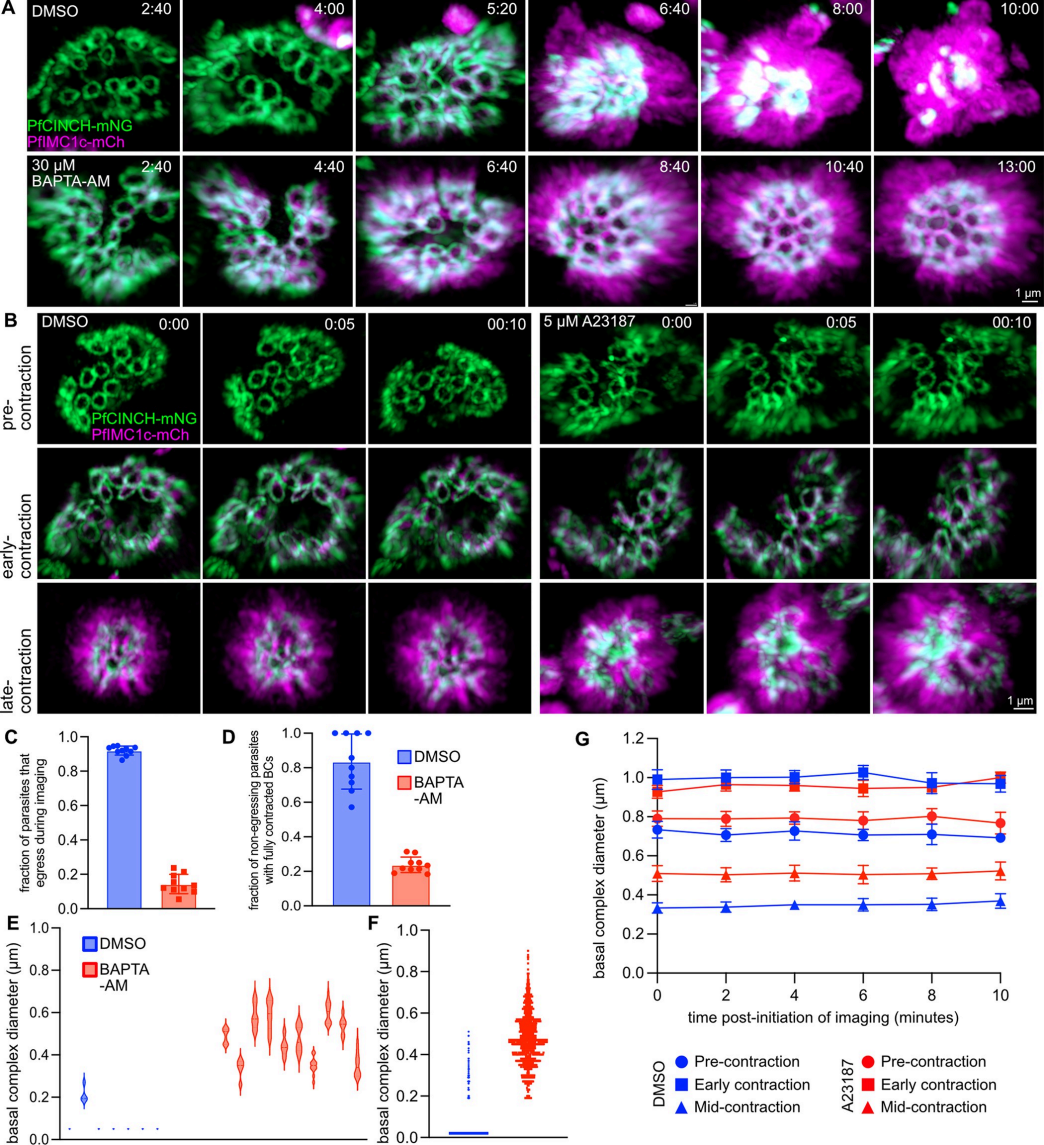
Calcium signaling is required, but not solely responsible for BC contraction. **A)** Selected time points of live cell imaging of PfCINCH-mNG; PfIMC1c-mCherry (mCh) parasites treated with DMSO (from Video 9, top row) or 30 μM BAPTA-AM (from Video 10, bottom row). **B)** Selected time points of live cell imaging of PfCINCH-mNG; PfIMC1c-mCherry (mCh) parasites starting at different stages of schizogony: pre-contraction (top row), early-contraction (middle row), and late-contraction (bottom row). Left panel shows parasites treated with DMSO, right panel shows parasites treated with 5 μM A23187. **C)** Fraction of parasites per field that egressed during imaging. **D)** Fraction of non-egressing parasites that had fully contracted basal complexes (BCs that contracted to the point that their diameter could not be measured) at the end point of imaging between DMSO and 30 μM BAPTA-AM-treated parasites. The data in **C)** and **D)** are displayed as mean ± SD with individual values overlayed. **E)** Plot of measured basal complex diameter (BCD) at the end point of imaging for all non-egressing parasites in a DMSO treated field and 10 selected non-egressing parasites in a BAPTA-AM treated field. Each violin = measurements from one parasite. **F)** Plot of measured BCDs from all non-egressing parasites in each DMSO-treated field and 10 selected non-egressing parasites from each BAPTA-AM treated field. For C-F, n = 10 fields/condition, 50–70 parasites/field. **G)** Graph of measured BCD for parasites in **B)** over the course of imaging. Conditions are matched by color, stage of segmentation is matched by symbol. Time depicted as hours:minutes. All scale bars = 1 μm.

Previous studies hypothesize that TgCen2 exerts contractile force on the *T*. *gondii* basal complex in a calcium-mediated manner, as centrins form calcium-responsive contractile fibers in green algae that are essential for multiple cellular processes in these organisms [26,60–64]. To test if this mechanism is conserved in *Plasmodium*, we performed long-term live-cell imaging in the PfCINCH-mNeonGreen; PfIMC1c-mCherry dual tagged line with a membrane-permeable calcium chelator, 30 μM BAPTA-AM [65]. Corroborating our PfCen2 iKD data, we saw chemical inhibition of contraction upon calcium chelation, although the IMC still formed normally, and the initial steps of BC formation and expansion were not hindered. No inhibition was observed in the control DMSO-treated condition (Fig 9A, S9–S10 Videos).

We quantified the contraction defect seen upon addition of BAPTA-AM in multiple ways. First, it has long been established that egress from the red blood cell is a calcium-dependent process and BAPTA-AM has previously been used to block egress by chelating calcium [65–69]. Thus, we first quantified the percentage of parasites which egressed over the course of imaging in each condition. 92±3% of parasites treated with DMSO egressed successfully, which speaks also to the general health of the imaged parasite population (Fig 9C). In contrast, only 14±6% of parasites egressed when treated with BAPTA-AM, and the presence of parasites which egress at all is likely due to the inclusion of parasites late enough in schizogony that their egress process could not be halted by chelating calcium (Fig 9C). We then examined all the parasites that did *not* egress and examined their basal complexes at the end point of imaging (Fig 9D). Of parasites that did not egress, 84±16% of DMSO-treated parasites had fully contracted basal complexes, whereas only 23±5% of BAPTA-AM treated parasites did (Fig 9D). Though the majority of BAPTA-AM treated parasites failed to fully contract their BCs, the BAPTA-AM treated parasites that had fully contracted basal complexes were likely those that were too advanced in contraction for the process to be halted by calcium chelation at the time imaging began. Thus, these data suggest that calcium chelation inhibits basal complex contraction.

Next, we measured the basal complex diameters of parasites which did not egress in both conditions. Because most of the non-egressing DMSO treated parasites had fully contracted BCs, we arbitrarily set those measurements to 0.05 μM to graph this data alongside data from BAPTA-AM treated parasites. Fig 9E shows a comparison of two fields, one treated with DMSO and one with BAPTA-AM: the average BCD for 10 BAPTA-AM-treated parasites in this field ranged from 0.35±0.06 to 0.60±0.05 μm and the mean BCD for the entire field was 0.48±0.10 μm (Fig 9E). In comparison, only one of the 7 non-egressing DMSO-treated parasites did not have fully contracted BCs, and this parasite had started imaging well before the BC was visible (Fig 9E). Overall, the mean BCD of BAPTA-AM treated parasites (at least ten BCs of ten schizonts in each of ten BAPTA-AM treated fields were measured, for a total of >1100 values) was 0.47±0.12 μm (Fig 9F). Interestingly, the vast majority of parasites began the process of imaging before BC contraction and yet were not completely defective in BC contraction. The max BCD of unexpanded *P*. *falciparum* parasites is around 1 μm, so BAPTA-AM treated parasites were able to contract down to just below half of their maximal diameter. Therefore, calcium signaling is required specifically for the latter stage of BC contraction and in the absence of intracellular calcium, the BC cannot contract to less than half of its maximal diameter [18].

This observation led us to wonder if calcium stimulation could induce contraction of the BC on its own, as had been shown in *T*. *gondii* [26]. We first tested the calcium ionophore, A23187, in a transgenic parasite line containing the ultrasensitive calcium sensor jGCaMP7c, a fusion of circularly permutated GFP, calmodulin, and a calmodulin-binding sequence (S9A and S9B Fig) [70]. After verifying its efficacy, we treated the PfCINCH-mNeonGreen; PfIMC1c-mCherry fluorescent line with 5 μM A23187 or DMSO and imaged parasites for 10 minutes, with images taken every minute (Fig 9B). Regardless of the stage of schizogony in which a specific parasite began imaging, there was no consistent, measurable decrease in BCD in either the DMSO or A23187 treated condition (Fig 9B and 9G). We tried extending the duration of imaging to 20 minutes and the interval between images to 2.5 minutes, but prolonged exposure to the calcium ionophore A23187 beyond this time frame resulted in schizonts which swelled and died, as previously reported, with no change in BCD [65] (S9C–S9D Fig). This is a striking departure from *T*. *gondii*, where treatment with 5 μM A23187 results in immediate, swift contraction within minutes. Thus, the presence of intracellular calcium is necessary, but not sufficient for final contraction of the basal complex in *P*. *falciparum* asexual replication [26].

## Discussion

In this work we identify two distinct spatial subcompartments within the *P*. *falciparum* basal complex. The first, more apical, contains PfCINCH and is termed the main ring. The second is more basal, appears as a diffraction-limited spot by standard immunofluorescence at the end of segmentation, and is termed the posterior cup. The posterior cup so far contains two basal complex proteins, PfSLACR and PfMyoJ. These two proteins also have similar temporal dynamics. In *Toxoplasma*, basal complex proteins which localize to the posterior cup (TgMyoJ, TgCen2, TgBCC1) also tend to be recruited to the basal complex after its initial formation [21,25,26]. It is interesting that the expression pattern and localization of PfMyoJ in the asexual replicative stage more closely resembles TgMyoJ than PbMyoJ: in *Plasmodium berghei*, PbMyoJ was not detected in the schizont stage, despite transcriptional data suggesting otherwise [40,71].

In *Toxoplasma*, 4 subcompartments have been identified: the basal pole/basal ring (containing most identified basal complex proteins including TgMORN1 and TgMyoC), the posterior cup, containing TgMyoJ, TgCEN2, TgBCC1, and TgBCC5, a compartment apical to the basal pole containing BCC2, BCC6, BCC7, and a compartment below the posterior cup, containing BCC8, BCC10, and BCC11 [22]. Identification of additional basal complex proteins and improvements in microscopy, such as a combination of U-ExM with stimulated emission depletion (STED) or iterative U-ExM may be required to further illuminate additional complexity which may exist in the *P*. *falciparum* basal complex [72,73]. The basal complex in *Plasmodium* could also be structurally simpler than in *T*. *gondii*, as is the case with the *Plasmodium* conoid [35,74].

Upon initial recruitment of PfMyoJ and PfSLACR to the basal complex at 46 HPI, both proteins nearly colocalize with PfCINCH, but both proteins localize to a more basal subcompartment of the BC over the course of contraction, suggesting that distinct subcompartments differentiate late in schizogony. *T*. *gondii* basal complex protein TgCen2, while also recruited to the basal complex at maximum BC expansion, is, in contrast, immediately localized to a subcompartment basal to TgMORN1 [26]. Either the *Toxoplasma* BC obtains spatial complexity earlier than the *Plasmodium* BC or visualization of earlier tachyzoites is needed to see colocalization of posterior cup proteins with TgMORN1. Indeed, while performing live-cell microscopy using our PfCINCH-mScarlet; PfMyoJ-mNeonGreen and PfCINCH-mScarlet; PfSLACR-mNeonGreen lines, careful analysis of schizonts developing in real time demonstrated that PfSLACR appeared at the basal complex slightly earlier than PfMyoJ. PfSLACR was recruited to the BC when the BCD is around 0.8 μm, while PfMyoJ is only significantly recruited when the BCD is around 1.0 μm, at its widest point. Thus PfSLACR, rather than PfMyoJ, is likely among the proteins which initially define the posterior cup subcompartment.

U-ExM demonstrated that both PfSLACR and PfMyoJ are unevenly removed from merozoites at the end of segmentation, a phenotype which indicates that *Plasmodium* merozoites produced from one schizont are not identical, and thus that segmentation is not entirely radially symmetric. The strands and agglomerates of PfSLACR and PfMyoJ departing from the basal complex colocalize, and live-cell microscopy confirms that this removal is simultaneous with colocalization in agglomerates and strands in the center of the parasite, likely in the residual body. In *T*. *gondii*, the residual body is an active organelle with an essential role in asexual replication [75]. Some proteins are transported to the residual body to perform a different function as endodyogeny nears completion [25,75]. TgCSAR1, for example, initially localizes to the maternal cytosol but is brought to the residual body during cytokinesis and is required for recycling the maternal cytoskeleton during cytokinesis [75]. Synchrony of tachyzoite division relies on active trafficking of cytosolic components between parasites along a network of actin filaments in the residual body [75]. TgMyoI also localizes to the residual body and its knockout results in a phenotype similar to ΔTgMyoJ, with disruption of this intercellular connection between tachyzoites [25]. While TgMyoI accumulates around the basal complex in extracellular tachyzoites, in ΔTgMyoJ parasites TgMyoI localizes to the basal complex exclusively [23,25]. TgMyoI could localize to the BC *before* transport to the residual body, like PfMyoJ/PfSLACR, with ΔTgMyoJ parasites lacking the basal complex maturation required for TgMyoI deposition at the residual body. The role of TgMyoI, which lacks a *Plasmodium* homolog, could have been taken up by PfMyoJ or PfSLACR which, like TgMyoI, has a coiled-coil domain, in *Plasmodium* asexual schizogony [25,76].

Following detailed characterization of the localizations of PfSLACR and PfMyoJ, we attempted preliminary functional characterization. Our attempts to knockout PfMyoJ were readily successful, which is not surprising because PbMyoJ has been knocked out in *P*. *berghei* and was predicted to be dispensable based on genetic screen data before that [39–41]. On the other hand, our previous localization of PfMyoJ to the basal complex in schizonts differed from the *P*. *berghei* data, where PbMyoJ was not seen in asexual stages, so it is interesting that PfMyoJ is both dispensable for and expressed in asexual blood stages [18,40]. ΔPfMyoJ parasites had no defect in replication or basal complex contraction, unlike ΔTgMyoJ tachyzoites, suggesting BC contraction occurs by different mechanisms in the asexual replicative cycles of *Plasmodium* and *Toxoplasma* [25]. Notably, the deletion of PfMyoJ and PfMORN1 together, both proteins with *T*. *gondii* homologs that are important for *T*. *gondii* BC function, still did not induce a defect in *P*. *falciparum* BC contraction [19,25,27,43]. Our initial attempts to knockout PfSLACR were unsuccessful. Although we did generate an inducible knockdown of PfSLACR using the same TetR-DOZI system that allowed us to effectively knockdown down PfCen2, there was no quantitative or qualitative fitness defect. Thus, characterization of the functional role of PfSLACR will likely require an inducible knockout.

We found that not only is the role of PfMyoJ and TgMyoJ different, but also that the role of actin filaments in BC contraction also differs between *T*. *gondii* and *P*. *falciparum*. When *T*. *gondii* tachyzoites are treated with 1 μM cytoD their basal complexes fail to contract and TgCen2 staining is lost [25]. However, cytoD treatment did not inhibit *P*. *falciparum* BC contraction, nor did it induce disorganization of merozoites as is seen in prolonged treatment of tachzyoites with cytoD [25]. The actin depolymerization inhibitor jasplakinolide also did not prevent BC construction, formation, or contraction despite both drugs readily preventing parasite reinvasion, as expected [47–49]. This indicates that there are major differences in basal complex constriction, particularly in force generation and contraction mechanisms, between *Plasmodium* and *Toxoplasma*, at least in the asexual stage of *T*. *gondii* development [77,78]. Importantly, our ability to visualize dual-fluorescently tagged parasites over the course of schizogony with no significant consequences for parasite development or morphology establishes a versatile live-cell imaging platform for testing whether genetic or chemical perturbations impact BC growth, expansion, contraction, and IMC connection with precise temporal control. We demonstrated the utility of this platform by applying compounds that disrupt actin dynamics right before imaging, allowing us to determine the role of dynamic actin filament formation in basal complex contraction specifically, rather than inducing actin excision at the ring stage and visualizing schizonts that have been actin deficient since the beginning of the erythrocytic growth cycle, as has previously been done.

We did identify one mechanistic similarity between *T*. *gondii* and *P*. *falciparum* BC contraction in asexual replication—namely the utilization of Cen2 and calcium-responsiveness [26,28]. Using an episomally-expressed PfCen2 under the control of its native promoter, we identified PfCen2 as a member of the basal complex, and a member of the basal cup in particular, a localization that aligns with that of TgCen2 [26,28]. Knockdown of endogenous PfCen2 demonstrated that PfCen2-deficient merozoites have larger basal complexes on average, but that BC contraction is not wholly abrogated. Much like TgCen2, PfCen2 seems to be required for the completion, but not the initiation of BC contraction [25]. Further, the fact that there is significant variance in the diameter of PfCen2-deficient parasite basal complexes indicates that PfCen2 may also be involved in ensuring synchrony of BC contraction during schizogony. This variance could also be due to either an incomplete knockdown or the fact that additional proteins are involved in BC contraction. Combining multiple knockdown and or knockout technologies and managing to attach an epitope tag to the endogenous PfCen2 locus would be of great use in answering this question.

Corroborating our evidence that PfCen2 is important for BC contraction but not solely responsible for the process, we made use of our live-cell imaging platform to show that chelating free calcium with membrane-permeable calcium chelator BAPTA-AM prohibited contraction of the BC below about half of its maximal diameter. However, unlike in asexual endodyogeny of *Toxoplasma*, treatment with calcium ionophore A23187 did not induce premature BC contraction or advance contractile processes already underway in our parasites, demonstrating that calcium signaling is necessary for BC contraction in *Plasmodium*, but not sufficient [26]. While calcium influx alone could potentially induce contraction on a longer time scale than our duration of imaging for these tests, after 20 minutes of imaging A23187-treated schizonts often swelled and died, as has been seen when calcium ionophores are added to immature (i.e., not immediately about to egress) *P*. *falciparum* schizonts [65,79–82]. One hypothesis to align these results is that calcium signaling is required for basal complex contraction but acts as a secondary signal rather than a primary driver of the contractile process. However, because PfCen2-deficient parasites have enlarged basal complexes, it remains likely that calcium-mediated contraction of PfCen2 is one of the force-generating mechanisms required in BC contraction, as is hypothesized for the contractile role of TgCen2 [26]. Thus, the lack of A23187-induced contraction likely indicates additional regulation of BC contraction in *Plasmodium* to maintain synchrony of BC contraction and merozoite development, a concern less relevant to the asexual replicative stage of *Toxoplasma*. Although, it would certainly be worthwhile to compare TgCen2-deficient parasites in the endodyogenic division stage that occurs within the cat, when synchrony is likely more relevant, to our PfCen2-deficient schizonts.

Functionally, these data suggest that in contrast to *T*. *gondii*, where TgMyoJ, actin, and TgCen2 work to generate force and induce final closure of the BC, only PfCen2 (and not PfMyoJ or PfActin) acts as a force-generating protein within the *P*. *falciparum* basal complex, and its final closure of the BC is dependent on free calcium [6,24–26]. It is known that centrins contain 4 calcium-binding EF-hands, and that the binding of calcium by these domains results in a conformational change, revealing hydrophobic residues that can participate in protein-protein interactions [83]. Both HsCEN2 and TgCen1 have been experimentally demonstrated to undergo polymerization/ self-assembly in a calcium-dependent manner [83–85]. Some of the earliest studies on centrins demonstrate that the contractile activity of centrin-based fibers is required for processes like flagellar excision and basal body reorientation in *Chlamydomonas* green algae [60–62,64,86]. It has been shown that the positively-charged N-terminal domain preceding the EF-hands is required for self-assembly in both human centrin 2 and *T*. *gondii* centrin 1 [85,87–89]. This evidence suggests a mechanism of action for PfCen2 in *Plasmodium*: self-assembly into calcium-dependent contractile fibers, with levels of regulation preventing premature contraction. With our combination of an inducible knockdown system and episomal constructs driven by the endogenous PfCen2 promoter, it could be possible to use the episomal constructs to complement the knockdown, and thus to test whether abrogating this N-terminal domain in the episomal copy would prohibit its ability to complement, in order to verify that self-assembly of PfCen2 into calcium-responsive fibers is required for its basal complex contractile activity.

Previous studies have demonstrated an important role for actin in cytokinesis [51,90]. Specifically, these studies found that depletion of actin or the actin-nucleating factor, formin-2, led to defects in apicoplast segregation and cytokinesis. In the light of our current study, we interpret these previous studies somewhat differently. We hypothesize that the defect in apicoplast fission and segregation, caused by depletion of actin or actin polymerization, causes a secondary defect in cytokinesis. The presence of incompletely segregated apicoplast (or other organelle) prevents successful basal complex-mediated cytokinesis by impairing its complete contraction. This is supported by multiple lines of evidence. In PfActin-deficient parasites, about 50% of the E64-stalled schizonts did not exhibit conjoined merozoites [90]. Even in schizonts with conjoined merozoites, some merozoites separated from the schizont mass, and though basal complex diameter was not measured, these released merozoites were of regular size and shape, making it likely they had fully contracted basal complexes, unlike merozoites generated from PfCen2-deficient schizonts in this study or TgCen2/TgMyoJ deficient tachyzoites in previous *Toxoplasma* work [25,26,28,90]. While our PfCen2-deficient schizonts did produce some merozoites with fully contracted BCs, enlarged-BC merozoites were found among both released merozoites and those that did not separate from the schizont, unlike PfActin-deficient parasites [90]. The authors also demonstrated an apicoplast-fission defect in PfActin-deficient parasites: apicoplasts in PfActin-deficient schizonts can form the large, tubular structures during early schizogony, but they collapse in the center of the parasite instead of dividing evenly among new merozoites, which is phenocopied in our work when cytochalasin D is added to schizonts [90].

This apicoplast segmentation phenotype and the failure to contract BCs only among merozoites that did not separate from the mass may instead be secondary to uncleaved apicoplast material occluding the basal complex, preventing full contraction and separation of many merozoites. We, therefore, hypothesize that actin primarily functions to separate the apicoplast during late schizogony and plays a minimal role in the formation, expansion, and contraction of the basal complex. This hypothesis also partially explains the actin chromobody data in the second study [51]. In this work, actin does not seem to localize to the basal complex throughout schizogony, as one would expect if it were a significant component of the basal complex. Instead, it only localizes to the basal complex in late schizogony once basal complex contraction has begun [51]. This observation does make sense if actin primarily serves to segregate the apicoplast, which is divided among merozoites in late schizogony well after contraction of the basal complex has begun [51,91].

It is also possible that the initial stages of BC contraction are driven not by energy-dependent, force-generating proteins we have not yet identified, but by energy-independent processes. As an explanation for how this might function, the “rubber band” model seems most attractive [21]. In this model, the stability of an expanding basal complex ring is most important and force is applied during the constructive phase to continually increase the BC’s diameter such that extensive force generation in the contractile stage is not necessary–a signal that marks the end of the BC expansion stage could induce contraction by initiating the release of this stored tension [21]. Strengthening this hypothesis, in both organisms, proteins essential to maintain BC stability have been identified, and the phenotypes of their depletion look remarkably similar although they are not structurally similar [18,21,23]. In addition, the BC in *T*. *gondii* recently has been suggested to serve as a ‘docking site’ for the addition of cytoskeletal components through alveolar vesicles [23]. Thus, the addition of lipids and proteins to the IMC and the gradual formation of this structure could itself force the BC to constrict, with only the final stage of contraction requiring the action of TgMyoJ, TgCen2, and actin, or PfCen2 and additional unknown players in schizogony, depending on the organism.

Of course, both our work and any comparisons to *T*. *gondii* cell division are necessarily limited to the asexual replicative stage, in which these two organisms differ significantly. While *T*. *gondii* does undergo endopolygeny during asexual development in the cat intestine in preparation for sexual differentiation, the difficulty, expense, and limitations of working with this stage as well as with the sexual stages of *P*. *falciparum*, have resulted in a focus on the asexual replicative stages of both parasites, despite the fact that this is the most significant point of divergence between their division processes [6,92–94]. Basal complex or IMC proteins have, however, previously been identified in both *T*. *gondii* and *Plasmodium* definitive host stages. TgMORN1 was identified in the asexual and sexual replicative processes of *T*. *gondii* in the intestinal cat epithelium, although the essentiality of TgMORN1 to this process was not determined [44]. IMC-associated alveolin proteins PbIMC1a, c, e, and h were identified in segmenting oocysts of *P*. *berghei*, indicating that the IMC and likely the BC are conserved in the process of sporozoite formation from the oocyst [95–98]. However, no similar work has been performed to examine BC and IMC protein localization in *P*. *falciparum*. Now that sexual differentiation of *T*. *gondii in vitro* has been made possible, much more thorough comparisons of division mechanisms between these species can be made as sexual replication can be examined in more thorough detail [95]. Requirements for synchrony that may drive the development of differences in asexual division mechanisms between asexual replicative mechanisms in *Plasmodium* and *T*. *gondii* may indeed be present in the endopolygenic replicative processes of *T*. *gondii* in the cat intestine.

Similarly, whether the dispensable proteins PfMORN1 and PfMyoJ are required for the development of other *P*. *falciparum* stages is an open question. Unlike *P*. *falciparum* MyoJ, *P*. *berghei* MyoJ was only visible in mature oocysts [40]. Its localization at the junction between developing sporozoites and the oocyst body indicates that this stage of *Plasmodium* development utilizes a basal complex-like structure, although PbMyoJ is not required for the development of invasive sporozoites [40]. While we did not test if our specific ΔMyoJ parasites are capable of generating invasive sporozoites, it would be interesting to determine if PfMyoJ, unlike PbMyoJ, is required for the maturation of ookinetes, oocysts, and sporozites [42].

Our data suggest that the actin intensities previously seen at the basal complex or at the basal edge of the IMC likely do not indicate a role for actin filaments in the construction or contraction of the basal complex, as both processes are carried out effectively in cytoD-treated parasites, thus further separating *P*. *falciparum’s* cytokinetic mechanisms from those of other eukaryotes [51,99]. Instead, we propose a mechanism of basal complex contraction based on calcium-dependent contraction, potentially via PfCen2 fibers, in conjunction with yet-unidentified proteins. This study identifies, for the first time, a mechanism of action for BC contraction in *P*. *falciparum*. Though incomplete, it represents a significant step forward and lays important groundwork for further investigation of this unique molecular machine, which could prove to be a useful target for novel antimalarials.

## Methods

### Plasmid construction

#### PfCINCH-smMyc (pAM27)

SmMyc was PCR amplified with oJDD4293/4925 and cloned as a NcoI/XmaI fragment into pRR206 [18]. The hDHFR cassette in this intermediate plasmid was removed by excision with AflII/AvrII and replaced with a blasticidin-S-deaminase (BSD) expression cassette. The PfCINCH-targeting regions from pRR92 [10] were excised with NotI/NcoI and cloned into the second intermediate plasmid using the same sites.

#### PfMyoJ-pFalKO (pAM135)

The homologous regions of PfMyoJ were amplified with oJDD6800/6801 (5’ HR) and oJDD6804/6805 (3’ HR). The hDHFR cassette and the pGEM backbone were amplified from pRR206 [18] using primers oJDD6798/6799 (pGEM backbone) and oJDD6802/6803 (hDHFR). All pieces were ligated using the BsaI GoldenGate kit (New England Biolabs).

#### PfMORN1- ^loxP^smV5 -BSD (pAM163)

The drug cassette containing BSD-resistance ORF was amplified from pAM27 [18] using primers oJDD7226/7227 and cloned as a BamHI/HindIII fragment into pCJM17 [19] digested using BamHI and HindIII.

#### PfMyoJ-mNeonGreen (pAM174)

The homologous regions of PfMyoJ were amplified with oJDD7203/7317 and cloned as a NotI/NcoI fragment into pAM166 [18] digested using NotI/NcoI.

#### PfSLACR-mNeonGreen (pAM175)

The homologous regions of PfSLACR were amplified with oJDD7213/7318 and cloned as a NotI/NcoI fragment into pAM166 [18] digested using NotI/NcoI.

#### PfSLACR-mCherry (pAM192)

mCherry was amplified using oJDD7736/7737 from AM110 and cloned as a NcoI/KpnI fragment into pAM160 [18] digested using NcoI/KpnI. Into this intermediate plasmid (digested with AflII/AvrII), the drug cassette containing hDHFR-resistance ORF was amplified from pRR206 [18] using primers oJDD7781/7782 and cloned as an AflII/AvrII fragment.

#### PfMyoJ-smMyc (pAM185)

The homologous regions of PfMyoJ were amplified with oJDD6358/7466 and cloned as a NotI/NcoI fragment into pAM159 [18], digested with NotI/NcoI.

#### PfIMC1c-mCherry (pAM179)

The homologous regions of PfIMC1c were amplified with oJDD7401/7402 from pAK67 and cloned as a NotI/NcoI fragment into pAM110 [18], digested with NotI/NcoI.

#### 2HA-PfCen2 (Ep) (pAM197)

The 5’ and 3’ UTRs flanking PfCen2 were amplified with oJDD7750/7751 (5’UTR) and oJDD7748/7749 (3’ UTR) from Pf3D7 genomic DNA. They were joined using PCR-SOE, then amplified using oJDD7748/8042, which appended a 2xHA tag 5’ to a synthesized codon-altered copy of PfCen2 (CaPfCen2), amplified with oJDD8043/8044. CaPfCen2 was then joined to the HRs using PCR-SOE and cloned as a NotI/KpnI fragment into pAM181 [18].

#### smV5-PfCen2 (Ep) (pAM203)

The 5’ and 3’ UTRs flanking PfCen2 were amplified with oJDD7748/oJDD8323 from pAM197. The 5’ UTR was joined using PCR-SOE to smV5, amplified from pAM145 with oJDD8324/8325. Then this piece was joined using PCR-SOE to the codon-altered copy of PfCen2, amplified with oJDD8326/8044. The final insert was cloned as a NotI/KpnI fragment into pAM197.

#### PfCen2^Tet^

The 3’ UTR flanking PfCen2 was amplified with oJDD7748/8568 from pAM197. The 5’ HR, now consisting of much of the protein sequence, was amplified with oJDD8569/8570, then oJDD8569/8571 to insert 75 base pairs of codon-altered PfCen2. The two HRs were then joined using PCR-SOE and cloned as a NotI/PspOMI fragment into Tet-aptamer and DOZI-cassette containing plasmid pPG03 [58].

#### jGCaMP7c (pIA06)

The pGEM backbone was amplified with from pJDD349 [58] with oJDD6272/6273. The BXB1 integrase, hDHFR cassette, and hsp86 promoter were amplified from pRLC98 [20] with oJDD6274/6279 and oJDD6278/6271, and the jGCaMP7c sequence was amplified from Plasmid #105321 from Addgene with oJDD6280/6277 (70). Pieces were assembled using the NEBridge Golden Gate Assembly Kit (BsmBI-v2) from New England Biolabs, Cat #e1602.

#### SpCas9 targeting plasmids

Guide oligos targeting specific genes were cloned into a lab generated Cas9 plasmid containing the P. falciparum U6 cassette, with *Bbs*I sites for guide oligo cloning located between the U6 promoter and the scaffold guide RNA [10]. Aside from the U6 cassette, this plasmid contains a 3xFLAG -tagged copy of SpCas9 under control of the *P*. *falciparum* hsp86 promoter and is followed by the PbDT terminator. For positive and negative selection, this plasmid contains the yeast Ura1 and FCU cassettes, respectively.

For tagging PfCINCH, PfSLACR, PfMyoJ, and PfMORN1, the guide oligos and plasmids have been described [10,18,19]. For knocking out PfMyoJ, oligos oJDD6806/6807 and oJDD6808/6809 were cloned into pRR216 to generate pAM161 and 162, respectively. For endogenously tagging PfCen2, oligos oJDD7755/7756 were cloned into pRR216 to generate pAM193.

### Reagents and antibodies

All primers were obtained from IDT (Integrated DNA Technologies) and their sequences are listed in S1 Table. All restriction enzymes were purchased from New England Biolabs. Mouse anti-V5 and mouse anti-Myc clone 9e10 antibodies were purchased from Bio-Rad. Rabbit anti-Myc polyclonal was purchased from Sigma Aldrich. Rabbit anti-PfGAP45 antibody was generously provided by Julian Rayner at the Cambridge Institute for Medical Research. Rabbit anti-PfMORN1 and anti-PfIMC1g were previously generated in the lab [10,15]. Mouse anti-MSP1 was generously provided by Anthony Holder at the Francis Crick Institute.

### *P*. *falciparum* culture

The *P*. *falciparum* 3D7 laboratory strain, obtained from the Walter and Eliza Hall Institute (Melbourne, Australia) was the basis for most transfections. The ΔPfMyoJ, and ΔPfMyoJ; PfMORN1- ^loxP^smV5 parasite strains were generated in the 3D7*pfs47*DiCre (referred to as 3D7-DiCre) background [42]. Parasites were cultured in RPMI-1640 (Sigma) supplemented with 25 mM HEPES (4-(2-hydroxyethel)-1-piperazineethanesulfonic acid) (EMD Biosciences), 0.21% sodium bicarbonate (Sigma), 50 mg/l hypoxanthine (Sigma), and 0.5% Albumax II (Invitrogen). Packed RBCs were obtained from Valley Biomedical. Parasites were cultured at 37°C with a gas mixture of 5% CO2.

### *P*. *falciparum* transfection

For all transfections except those used to create the 2HA-Cen and smV5-Cen2 episomally maintained lines (AM197, AM203) and the jGCaMP7c line (IA06), 25 μg of HDR plasmid was linearized by digestion, purified, and co-transfected with 20 μg of the relevant guide plasmid containing an SpCas9 expression cassette and the PfSLACR-targeting guide RNA into the relevant parental *P*. *falciparum* strain, synchronized as schizonts using the Amaxa 4D system. One day post transfection, drug pressure was applied with the relevant drug. Details for all transfections (plasmids, guide plasmids, and parental strains utilized) can be found in S2 Table. For creating 2HA-Cen2 and smV5-Cen2 (Ep) lines, transfection procedures were identical except the plasmids were not linearized beforehand. For creating the jGCaMP7c strain, 100 μg of the plasmid (IA06), which contains an attP sequence, the mycobacteriophage-derived Bxb1 integrase under control of the *P*. *falciparum* CAM promoter, and the jGCaMP7c expression cassette was transfected into a *P*. *falciparum* strain containing a markerless insertion of the attB sequence in the cg6 locus [100].

### Replication assay

Synchronized 3D7-DiCre, ΔPfMyoJ, and/or PfMORN1- ^loxP^smV5 (± rapamycin), or PfCen2^Tet^ (± ATc) was diluted to 0.2% parasitemia and 1% hematocrit and plated in triplicate. 100 μL of culture from each well was collected on days 1, 3, 5, and 7, washed with PBS, and resuspended in a 1:1000 dilution of SYBR Green I (S7563, Invitrogen) in 0.5% bovine serum albumin (BSA) in PBS. Parasites were incubated in the solution for 20 minutes at room temperature, then washed in 0.5% BSA in PBS and resuspended in filtered PBS. The proportion of infected cells was then determined by flow cytometry.

### Standard Immunofluorescence

If schizonts were stalled with C1 or E64, schizont pellets were percoll-purified and allowed to incubate in 5 mL of media with 2.5 uM C1 or 10 uM E64 for 2–3 hours depending on the desired stage of purified schizonts. These schizonts were then pelleted and immunofluorescence protocol was performed as described below. Schizont pellets from percoll-purified parasite cultures were resuspended in 100 μL PBS and placed on a poly-D-lysine coated #1.5 10mm coverslip, inserted into one well of a 24-well plate. Parasites were allowed to settle at 37C for 30 minutes. Excess media was then removed and 300 μL of 4% paraformaldehyde (PFA) in PBS was added to the side of the well, for 20 minutes of fixation also at 37C. PFA was then removed, and the coverslip was washed 3 times with PBS. Following fixation and washing, parasites were permeabilized with 0.1% Triton X-100, diluted in PBS, for 10 minutes, and washed again in 1X PBS, 3X for 3 minutes. Blocking solution (3% (w/v) BSA in PBS) was added to coverslips for 1 hour at room temperature or overnight at 4C. Primary antibodies were diluted in blocking solution and added to coverslips for 1 hour at room temperature or overnight at 4C. Following primary antibody incubation, coverslips were incubated 3X for 5 minutes in 1X PBS. Secondary antibodies, isotype-specific for dual mouse primaries, diluted in 0.5% BSA-PBS, were then added to coverslips for 45 minutes at room temperature. Coverslips were washed again for 5 minutes, 3X in 1X PBS, then incubated with Hoechst 33342 diluted at 1:5000 in PBS. After rinsing 3 more times with 1X PBS, coverslips were mounted with VectaShield Vibrance. Cells were visualized on a Zeiss LSM900 with Airyscan2 for super-resolution microscopy using a 63X objective with a numerical aperture of 1.4.

Dilutions for primary antibodies are: mouse anti-V5 1:500, rabbit anti-PfGAP45 1:5000, mouse anti-Myc 1:100, rabbit anti-Myc 1:1000, mouse anti-MSP1 1:500, rabbit anti-IMC1g 1:1000.

### Ultrastructure expansion microscopy

Synchronized schizonts were purified via density centrifugation with 60% Percoll. If schizonts were stalled with C1 or E64, schizont pellets were percoll-purified and allowed to incubate in 5 mL of media with 2.5 uM C1 or 10 uM E64 for 2–3 hours depending on the desired stage of purified schizonts. These schizonts were then pelleted and immunofluorescence protocol was performed as described below. Isolated schizonts were allowed to settle on poly-D-lysine coated coverslips placed into one well of a 24-well plate for 30 minutes at 37C. Parasites were fixed with 4% PFA for 20 minutes at 37C and washed 3X with PBS, and fixed slips were incubated with FA/AA overnight at 37C. Gel polymerization was performed the following morning, roughly 15 hours after FA/AA was added, on ice. TEMED and APS were added to a previously-made monomer solution and the coverslips were placed over a drop of the TEMED/APS/Monomer Solution mixture in a gelation chamber, which had been stored at -20C for at least 15 minutes before gelation began. After 5 minutes of incubation on ice, the chamber was incubated at 37C for one hour. Post incubation, coverslips and gels were placed in 1 mL of denaturation buffer in one well of a 6 well plate and incubated with denaturation buffer for 15 minutes with agitation. After gel detachment, gels were incubated for 90 more minutes in a 1.5 mL tube filled with denaturation buffer at 95C. After this incubation, denaturation buffer was removed and gels were placed in a 10 cm dish filled with ddH2O, which was switched out after 30 minutes. After overnight incubation in ddH2O, gels were washed in PBS 2 times for 15 minutes each then incubated in 3% BSA-PBS for 30 minutes at RT. Gels were then incubated in 1 mL of 3% BSA-PBS with primary antibodies (rabbit anti-Myc, 1:200; mouse anti-V5, 1:250; rabbit anti-IMC1g 1:500) overnight at 4C. The next morning, gels were washed 3 times with 2 mL 0.1% Tween20 in PBS for 10 minutes at room temperature with agitation. After washes, gels were incubated in 1 mL of PBS with secondary antibodies including AlexaFluor 405-NHS-Ester (1:100) and SYTOX Deep Red (1:1000), protected from light. After 2:30, gels were washed 3 more times with 2 mL 0.1% PBS + 0.1% Tween20 as previously, then placed in a 10 cm dish filled with ddH2O. Water was replaced after 30 minutes, and gels were allowed to expand overnight before imaging on a Zeiss LSM900 with Airyscan 2. This expansion protocol regularly provides 4–4.5-fold expansion [36]. To quantitatively determine the gel expansion factor, we either measured the size of the gel before and after expansion using a caliper or used organelles / structures of known size, such as the centriole, as a “molecular ruler” against which the size of the measured, expanded structure can be compared depending on the structures visualized.

### Live cell microscopy

All lines were prepared for imaging in the same way: parasites were synchronized as required for the experiment and percoll-purified at the schizont stage. Isolated schizonts were then allowed to recover in a 5 mL dish with complete media for 30 minutes. The dish was then spun down and the parasite pellet was resuspended in 100 μL media and allowed to settle on one quadrant of a concanavalin A-coated iBidi/cellview glass bottom dish. After thirty minutes, excess/unbound red blood cells were washed off with 3 washes of PBS, then each quadrant was filled with phenol-red free RPMI, with Trolox added to a concentration of 0.5 mM to minimize bleaching and oxidative damage.

For Figs 4A and 4B, S2C, S3A,S3B,S3E four fields were selected based on parasite density and stage of schizont. They were imaged every 3 minutes over 2.5 hours in the SR-Multiplex Mode (4Y), using the 488 laser at 0.9% laser power and 870 gain, and the 561 laser at 1% laser power and 850 gain.

For Fig 4E and 4F, four fields were selected based on parasite density and stage of schizont. They were imaged every 3 minutes over 2.5 hours in the Confocal Mode, using the 488 laser at 0.9% laser power and 870 gain, the 561 laser at 1% laser power and 850 gain, and the 647 laser through ESID to obtain brightfield/transmitted light imaging.

For Figs 5A–5D and S4A–S4B, eight fields were selected based on parasite density and stage of schizont. They were imaged every 15 minutes over 12 hours in the SR-Multiplex Mode (4Y), using the 488 laser at 0.9% laser power and 870 gain, and the 561 laser at 1% laser power and 850 gain.

For Figs 5E and S4G, both lines were amplified and then synchronized to within 2 hours. At 44 HPI, the aforementioned preparation for imaging was performed. Six fields per line were selected based on parasite density and stage of schizont (trying to maximize parasites around 44 HPI). All 12 fields were imaged every 20 minutes over 5 hours in the SR-Multiplex Mode, using the 488 laser a 0.9% laser power and 870 gain, and the 561 laser at 1% laser power and 850 gain.

For Figs 7, 9C, 9D, and S5B, 5–10 fields per condition were selected based on parasite density and stage of schizont. Drug or the equivalent volume of DMSO was added 30 minutes, 2 hours, or 4 hours before imaging depending on the experiment. They were imaged every 20 minutes over 13 hours in the SR-Multiplex Mode (4Y), using the 488 laser at 0.9% laser power and 870 gain, and the 561 laser at 1% laser power and 850 gain. For S5A Fig, 3–4 fields per condition were selected based on parasite density and stage of schizont. They were imaged every 20 minutes over 12 hours in the SR-Multiplex Mode (2Y), using the 488 laser at 0.9% power and 870 gain.

For Fig 9B, a single field of parasites per biological replicate was selected for imaging based on parasite density and the presence of multiple stages of schizont. A23187 or the equivalent volume of DMSO was added between the first and second time point. Parasites were imaged every minute or every other minute, depending on the experimental conditions, for 10–20 minutes, in the SR-Multiplex Mode (4Y), using the 488 laser at 0.6% laser power and 870 gain and the 561 laser at 0.8% laser power and 850 gain.

For all live cell experiments, parasites were imaged in a 37C incubation chamber supplemented with 5% CO2. They were visualized on a Zeiss LSM900 equipped with AiryScan2 processing using either the SR-Multiplex Mode (4Y or 2Y) or the Confocal imaging mode. For measurements, resulting files were processed in FIJI, to produce supplementary videos and high-resolution images, resulting files were processed in ARIVIS 4D.

### Image processing

#### Display

For most of the z-stack images taken on the Zeiss LSM900 with AiryScan2 and processed in ImageJ, each image was loaded into ImageJ and brightness and contrast were modulated solely by using the reset command (autoscale) on the brightness and contrast panel. This command restores the original brightness and contrast settings, setting the display range to the full pixel value of the image, and rendering processed images as similar as possible to native images. For certain images (listed in the relevant figures), the intensity values for each channel were matched to those of a previously ‘autoscaled’ parasite using the SET command on FIJI.

#### Measurement

For measurements of PfCINCH-PfMyoJ/PfCINCH-PfSLACR peak distance in Fig 2 (Fig 2C and 2D), rings were selected and measurement upon them was performed as depicted in S1 Fig. For ease of analysis, eight rings per schizont were selected for intra-ring relationship analysis and comparison, with only the NHS-Ester and PfCINCH channels visible, such that the position of PfMyoJ/PfSLACR relative to PfCINCH was not visible and did not bias the selection of rings. For larger rings (mid segmentation/early contraction), a Z-projection of the stacks across which the ring was visualized were generated and used as the basis of measurements. Lines along which fluorescence intensity for each protein were measured were drawn using the straight/segmented/freehand lines tool on FIJI. Each line crosses through the center of the ring and crosses the ring’s perimeter twice, resulting in two fluorescence “peaks” for PfCINCH generally. For *en face* or three quarters view rings both a horizontal and a vertical line were drawn and used to measure except when there was a gap in the fluorescence (an occasional experience when using U-ExM due to the distribution of antibodies). For the other measurement(s), additional lines were drawn at angles corresponding to the positions on a hypothetical clock face depending on where the strongest signal was.

Fluorescence intensity profiles along each line for each channel were obtained using the plot profile command on FIJI and exported as CSV-lists. The fluorescence intensity values for both channels were divided by the maximum value in the list for each lin, thus normalizing measurements. To determine the distance between two proteins in the basal complex, the difference between the x-coordinates (distance in microns) of adjacent PfCINCH and PfMyoJ or PfSLACR peaks (identified by the y-coordinates; for one set of peaks/one crossing of the perimeter this will be the place where y = 1, for the other peak it will be the position where y is nearer 1 than the point immediately before or after it) was determined. The calculated distance values for each line were then averaged. Averaged distances from individual rings were then graphed and compared to the data from a mid-segmentation (46 hpi) parasite. Using a one-tailed student’s T-test, it was determined whether the mean distance between two proteins was larger in late-segmentation than in mid-segmentation parasites, when PfMyoJ and PfSLACR are first recruited to the basal complex.

For the subfigures designating PfMyoJ and PfSLACR as more basal than PfCINCH (Fig 2E and 2F), an additional set of measurements was performed. When the *P*. *falciparum* schizont undergoes segmentation, for the majority of late segmentation (i.e., during contraction of the basal complex) the nascent merozoites are oriented in a ‘rosette’ wherein their basal ends–and thus their basal complexes—are all pointed inward toward the center of the parasite such that each ring is at an angle. There ended up being only two time points where we could confidently identify merozoites oriented near-perfectly horizontally such that the distance along the apical- basal axis between two rings of protein could reliably be measured: mid-segmentation and post-egress. Ten rings per schizont were selected in an unbiased way, with only the NHS-Ester and PfCINCH channels visible. Unlike our first analyses, only completely “sideways rings” were selected for this measurement process. For these subfigures, each line was drawn perpendicular to the line of the ring, determined by comparing the angle of the ring-portion to that of the drawn line. Two lines were drawn across the “sideways ring”, preferably on opposite sides of the ring unless uneven fluorescence made that impossible. Lines were 1.0 microns in length, and ALWAYS oriented apical to basal, using nascent apical organelles such as the rhoptries as markers of the parasite’s apical end so the more basal protein would always have its fluorescence peak associated with a greater distance along the line. Line drawing, fluorescence value acquisition and normalization, and distance calculation were performed as with the previous rings. After the distance between the two proteins at each peak in each linear profile was calculated, the two values were averaged. Again, using a one-tailed student’s T-test, it was determined whether the mean APICAL-BASAL distance between PfCINCH and PfSLACR is larger in post egress parasites than in mid-segmentation parasites when PfMyoJ and PfSLACR are first recruited to the basal complex.

For Fig 3G–3H, “SLACR negative” or “MyoJ negative” merozoites (in post-egress) / basal complexes (in pre-egress) were identified by comparing the intensity of fluorescence signal in the 488 channel for a given PfCINCH-smMYC/NHS Ester AF405-identified basal complex that seemed to lack visible PfSLACR or PfMyoJ when autoscaling was applied to that of an easily-identifiable PfSLACR/MyoJ “positive” BC in the same or an immediately adjacent Z-slice. If the seemingly negative basal complex ring had a fluorescence intensity ≤ 20% of the easily-identifiable ‘positive’ BC, it was counted as a PfSLACR- or PfMyoJ-negative merozoite. For S2E Fig, “SLACR negative” or “MyoJ negative” merozoites were identified, using the PfMyoJ-smV5; PfSLACR-smMyc line, by comparing the intensity of fluorescence signal in the 488 channel for a given NHS Ester AF405-identified basal complex that seemed to lack visible PfMyoJ when autoscaling was applied to that of an easily-identifiable PfMyoJ “positive” BC in the same or an immediately adjacent Z-slice. If the seemingly negative basal complex ring had a fluorescence intensity ≤ 20% of the easily-identifiable ‘positive’ BC, it was counted as a PfMyoJ-negative merozoite. The same process was repeated using the 555 channel to determine if the same PfMyoJ-positive or -negative merozoite was also PfSLACR-positive or -negative.

For Figs 4A–4D and S3C–S3D, a circular region of interest was drawn precisely around ten easily-identifiable/clearly separate merozoites present in the relevant parasite’s maximum-projection image using the circular draw tool. Each ROI was re-drawn at each time point so the fluorescence intensity of the same merozoite was tracked over time, from when individual merozoites were identifiable to the final time point before egress. The min&max grey value as well as the mean grey value were then measured using FIJI’s set measurement and measure tools; the mean grey value was recorded and divided by the original (TP0) mean grey value for each merozoite to determine relative change in fluorescence for each merozoite. To measure the change in fluorescence of the entire parasite, a rectangular region of interest was drawn over the entire schizont at its largest point—usually the immediately pre-egress time point. The same rectangular ROI was used to identify the mean grey value for the entire schizont at each time point, and as with the individual merozoites, the mean grey value for each later time point was divided by the original (TP0) mean grey value for the entire schizont to determine relative change in fluorescence.

For Figs 5C–5D, 9E–9G, and S4C–S4F and S4H, to generate measurements of ring diameter, each visible ring of the basal complex was traced using the FIJI freehand selection tool and an ellipse was fit to the freehand tracing using the set measurements and measure tools. The major diameter for each ellipse was reported. This process was repeated for each time point so changes in BCD could be determined over time for the parasites visualized in Figs 5A–5B, S4C–S4F, S4H, and was only performed on select time points for the majority of the measured schizonts (blue boxes in Fig 5C–5D).

For Figs 6C, 6G, 7H, and 8D 8–10 post-egress parasites were identified for each condition. The basal complex was identified as a protein density ring at the basal end of the merozoite and the diameter was measured by drawing a circle around the contracted basal complex ring and once again fitting an ellipse to the freehand tracing using the set measurements and measure tools. The major diameter for each ellipse was reported.

## Supporting information

S1 Fig Method of measuring distances between PfCINCH and PfSLACR/PfMyoJ. **A)** Example of PfMyoJ and PfCINCH dual tagged *en face* BC ring with lines drawn at 4 predetermined angles—U-ExM image (left) and diagram (right). **B)** Fluorescence intensity graphs overlaying PfMyoJ (green) and PfCINCH (magenta) intensity values, each corresponding with the relevant line in **A)**. Black bars between the different colored peaks represent the distance between these two proteins by comparing the distance between their highest intensities. **C)** Calculated distance for each peak intersection measurement corresponding to the graphs in **B)**. **D)** Example of PfMyoJ and PfCINCH dual tagged sideways BC ring with two perpendicular lines drawn across the ring from apical to basal in orientation. Top = mid segmentation U-ExM image (left) and model (right); Bottom = post-egress U-ExM image (left) and model (right). **E)** same as **B)** but for the lines drawn and labelled in **D)**. **F)** same as **C)** but for distances demonstrated in **D)** and **E)**. All scale bars = 1 μm.
(TIFF)

S2 Fig Additional images of PfMyoJ and PfSLACR non-basal complex localization. **A)** Immunofluorescence of two dual-tagged PfSLACR and PfMyoJ lines to show colocalization of proteins in extra-BC localizations, along with IMC protein PfGAP45. Left: PfSLACR-smV5, PfMyoJ-smMyc. Right: PfMyoJ-smV5; PfSLACR-smMyc. **B)** U-ExM slices of PfMyoJ-smV5; PfSLACR-smMyc parasites during late contraction, complete contraction, and post-egress (E64-stalled). **C)** Selected time points of live cell imaging of a PfMyoJ-mNeonGreen; PfSLACR-mCherry parasite. **D)** U-ExM slices of PfMyoJ-smV5; PfSLACR-smMyc parasites illustrating various non-basal complex localization phenotypes. **E)** Quantification of merozoite heterogeneity in post-egress PfMyoJ-smV5; PfSLACR-smMyc parasites. n = 8 schizonts with 25–37 merozoites/schizont per time point. **F)** Representation of dual positive and dual negative merozoite from the PfMyoJ-smV5; PfSLACR-smMyc parasite line. Both merozoites’ basal complexes were in the same focal plane. The data in **E)** are displayed as mean ± SD with individual values also presented. Time represented as hours:minutes. All scale bars = 1 μm.
(TIFF)

S3 Fig Additional replicates of live cell imaging demonstrating PfMyoJ and PfSLACR removal from the basal complex in late segmentation. **A)** Selected time points of live cell imaging of PfMyoJ-mNeonGreen; PfCINCH-mScarlet parasite starting immediately pre-egress. **B)** Same as **A)** but with PfSLACR-mNeonGreen; PfCINCH-mScarlet parasite. **C)** Plot comparing changes in the fluorescence intensity of individual merozoites (in the PfMyoJ Channel) of the schizont represented in **A)** over time relative to changes in the entire schizont. **D)** Same as **C)**, but with PfSLACR; graph made from data taken from schizont in **B)**. **E)** Selected time points of live cell microscopy of PfMyoJ-mNeonGreen; PfSLACR-mCherry parasite starting immediately pre-egress. **F)** Additional U-ExM image of PfMyoJ-smV5; PfSLACR-smMyc parasite where PfMyoJ-smV5 and PfSLACR-smMyc colocalize in extra-merozoite protein agglomerates. All times represented as hours:minutes. All scale bars = 1 μm.
(TIFF)

S4 Fig Additional replicates of live cell imaging demonstrating PfSLACR’s earlier recruitment to the BC. **A)** Selected time points of live cell live cell imaging of PfMyoJ-mNeonGreen; PfCINCH-mScarlet parasites. Green box = initial observation of contiguous PfMyoJ ring. Blue dashed box = time point when BC diameter is greatest. **B)** Same as **A)** but with PfSLACR-mNeonGreen; PfCINCH-mScarlet parasites. **C)** Graph of mean ring diameter of schizont in **A)** over time; pink line = measurements for schizont in **A)**. Green point = initial observation of contiguous PfMyoJ ring. **D)** Graph of mean ring diameter of schizont in **B)** over time; pink line = measurements for schizont in **B)**. Green point = initial observation of contiguous PfSLACR ring. **E)** Graph of mean ring diameter of multiple PfMyoJ-mNeonGreen; PfCINCH-mScarlet schizonts with clear, measurable rings over time; green points = initial observation of contiguous PfMyoJ ring in each parasite. **F)** Same, but with PfSLACR-mNeonGreen; PfCINCH-mScarlet schizonts. **G)** Selected time points from synchronized live cell microscopy experiment comparing BCD-matched PfMyoJ-mNeongreen; PfCINCH-mScarlet parasites (top two rows) and PfSLACR-mNeonGreen; PfCINCH-mScarlet parasites (bottom two rows). **H)** graph of mean BCD for parasites in **G)** over time, with the green point representing initial observation of contiguous ring of each protein. Data in **C)**, **D)**, **E)**, **F)**, **H)** represented as mean ± SD. Time represented as hours:minutes. All scale bars = 1 μm.
(TIFF)

S5 Fig PCRs confirming disruption of PfMyoJ and PfMORN1 loci. **A)** Diagram demonstrating ΔPfMyoJ creation strategy using double-crossover mediated integration and inducing a 217-base pair deletion between the HRs as well as the insertion of the donor cassette. **B)** Second replicate of flow cytometry-based replication curve comparing ΔPfMyoJ parasites to parental 3D7-DiCre line. **C)** Diagram comparing the PfMyoJ genomic locus upon integration of the disrupting HDR plasmid (dotted box) to the native genomic locus and integration PCRs for ΔPfMyoJ, confirming the disruption of this locus, with primers A & B, C & D, A & D or CTL primers (oJDD5078/5079 in. S1 Table). **D)** Diagram comparing the PfMORN1 genomic locus upon integration of the HDR plasmid (dotted box) to the native genomic locus as well as the integrated locus upon addition of rapamycin. Integration PCRs for PfMORN^loxP^-smV5, demonstrating successful excision of the majority of PfMORN1 in the bulk population as well as cloning of an excised dual-knockout parasite with primers E & F, G & H, E & H or CTL primers (oJDD5078/5079 in S1 Table).
(TIFF)

S6 Fig Additional cytochalasin D data confirming chemical disruption of actin dynamics does not inhibit BC contraction. **A)** Selected time points of live cell imaging showing basal complex contraction in PfCINCH-mNeonGreen, PfIMC1c-mCherry parasites treated with DMSO (top row) 1 μM (middle row) or 2 μM (bottom row) cytoD. **B)** Selected time points of live cell imaging showing basal complex contraction in PfCINCH-mNeonGreen; PfIMC1c-mCherry parasites treated with DMSO (top row) or 2 μM (bottom row) cytoD. **C)** Same as *b* but with 4 μM cytoD. **D)** Comparison of fraction of egressing parasites per field with fully contracted basal complexes (ie, BCs that contracted to the point that their diameter could not be measured) between DMSO, 1 μM, and 2 μM cytoD treated parasites depicted in **A)**. n = 3–4 fields/condition, 20–30 parasites/field. **E)** Comparison of fraction of egressing parasites per field with fully contracted basal complexes between DMSO and 2 μM cytoD parasites depicted in **B)**. n = 6 fields/condition, 30–50 parasites/field. **F)** Comparison of fraction of egressing parasites per field with fully contracted basal complexes between DMSO and 4 μM cytoD parasites depicted in **C)**. n = 6 fields/condition, 30–50 parasites/field. Time represented as hours: minutes. For **D)**-**F)**, data are displayed as mean ± SD with individual values overlayed. All scale bars = 1 μm.
(TIFF)

S7 Fig Experiments confirming cytochalasin D activity on apicoplast replication and merozoite invasion. **A)** Field’s stain images of resulting parasitemia taken 4 hours after percoll-purified schizonts were added to red blood cells with 2 μM cytochalasin D or an equivalent volume of DMSO were added. **B)** Quantification of ring parasitemia in wells imaged in **A)**. The data are displayed as mean ± SD with individual values overlayed; reinvasion assays were performed in triplicate and these data represent one of two biological replicates. **C)** Immunofluorescence of late schizonts treated with DMSO or 2 μM cytochalasin D comparing distribution of plasma membrane protein PfMSP1 and apicoplast protein PfACP.
(TIFF)

S8 Fig Verification of episomal PfCen2 expression and PfCen2^Tet^ integration. **A)** Immunofluorescence of episomally-expressed 2HA-PfCen2 in early schizogony, pre-cytokinesis, and post-egress (E64-stalled) with basal complex marker PfMORN1. **B)** U-ExM of episomally-expressed smV5-Cen2 and far-red DNA dye SYTOX in early schizogony, pre-cytokinesis, demonstrating localization of smV5-PfCen2 to the outer centriolar plaque. Red box indicates the 1.5x-zoomed region to the right of this panel, in which a red arrow points to smV5-PfCen2 localization in the centriolar plaque. **C)** U-ExM of episomally-expressed smV5-Cen2 and IMC-associated alveolin protein PfIMC1g in post-egress merozoites (from E64-stalled culture). Two different projections of 8–10 slices are shown; red arrows point to localization of smV5-PfCen2 to the basal cup in these mature merozoites. **D)** Diagram comparing the PfCen2 genomic locus upon integration of the disrupting HDR plasmid (dotted box) to the native genomic locus and integration PCRs for PfCen2^Tet^, confirming the disruption of this locus, with primers I & J, G & K, I & K or CTL primers (oJDD5078/5079 in S1 Table). **E)** Second replicate of flow cytometry-based replication curve comparing PfCen2^Tet^ parasites in the presence (PfCen2-sufficient) and absence (PfCen2-deficient) of ATc. **F)** U-ExM slices of additional post-egress (E64 stalled) PfCen2-deficient PfCen2^Tet^ parasite. Numbered, dashed boxes indicate the respective numbered 1.5x-zoomed region to the right of the panels; red, white, and yellow arrows point to enlarged basal complexes of various sizes and blue arrow indicates a normally sized basal complex. All scale bars = 1 μm except the non-zoomed U-ExM panels in **F)** where scale bar = 5 μm.
(TIFF)

S9 Fig Verification and additional testing of calcium ionophore A23187. **A)** Selected time points of live cell imaging showing a single slice of jGCaMP7c-expressing parasites after the addition of either DMSO or 5 μM A23187, validating the efficacy of this compound. Time represented as seconds post initiating imaging. **B)** Quantification of **A)**; mean fluorescence intensity of the parasite depicted in **A)** was calculated for each image taken at each 5-second interval and normalized to fluorescence intensity of the first image, immediately after initiating imaging. **C)** Selected time points of live cell imaging of PfCINCH-mNG; PfIMC1c-mCherry (mCh) parasites starting at different stages of schizogony: pre-contraction (top row), early-contraction (middle row), and late-contraction (bottom row) after the addition of 5 μM A23187, imaged for 20 minutes with 2.5 minute intervals. Time represented as hours:minutes. **D)** Graph of measured BCD for parasites in **C)** over the course of imaging. Stage of segmentation is matched by symbol. All scale bars = 1 μm.
(TIFF)

S1 Table Description: Primers used in this study. (XLSX)

S2 Table Description: List of transfections performed to generate the strains utilized in this study with parental line name, HDR plasmid name and description, SpCas9 + targeting guide plasmid name, paper wherein that targeting guide was first used, if relevant, drug used for selection, and drug used for maintaining parental line. (XLSX)

S1 Video Description: 3-Dimensional rendering of time-lapse confocal (AiryScan 4Y Multiplex) microscopy of PfCINCH-mScarlet; PfMyoJ-mNeonGreen parasites. PfCINCH-mScarlet is visible in magenta, PfMyoJ-mNeonGreen is visible in green. Leftmost panel is a merge of both colors, middle panel is PfCINCH-mScarlet only, rightmost panel is PfMyoJ-mNeonGreen only. Z-stacks were collected every 3.5 minutes for 3 hours; frame rate is 0.5 seconds per time point. Scale bar = 1 μm. Corresponds with Fig 4A.
(MP4)

S2 Video Description: 3-Dimensional rendering of time-lapse confocal (AiryScan 4Y Multiplex) microscopy of PfCINCH-mScarlet; PfSLACR-mNeonGreen parasites. PfCINCH-mScarlet is visible in magenta, PfSLACR-mNeonGreen is visible in green. Leftmost panel is a merge of both colors, middle panel is PfCINCH-mScarlet only, rightmost panel is PfSLACR-mNeonGreen only. Z-stacks were collected every 3.5 minutes for 3 hours; frame rate is 0.5 seconds per time point. Scale bar = 1 μm. Corresponds with Fig 4B.
(MP4)

S3 Video Description: 3-Dimensional rendering of time-lapse confocal (AiryScan 4Y Multiplex) microscopy of PfCINCH-mScarlet; PfMyoJ-mNeonGreen parasites. PfCINCH-mScarlet is visible in magenta, PfMyoJ-mNeonGreen is visible in green. Leftmost panel is a merge of both colors, middle panel is PfCINCH-mScarlet only, rightmost panel is PfMyoJ-mNeonGreen only. Z-stacks were collected every 15 minutes for 11 hours; frame rate is 0.5 seconds per time point. Scale bar = 1 μm. Corresponds with Fig 5A.
(MP4)

S4 Video Description: 3-Dimensional rendering of time-lapse confocal (AiryScan 4Y Multiplex) microscopy of PfCINCH-mScarlet; PfSLACR-mNeonGreen parasites. PfCINCH-mScarlet is visible in magenta, PfSLACR-mNeonGreen is visible in green. Leftmost panel is a merge of both colors, middle panel is PfCINCH-mScarlet only, rightmost panel is PfSLACR-mNeonGreen only. Z-stacks were collected every 15 minutes for 11 hours; frame rate is 0.5 seconds per time point. Scale bar = 1 μm. Corresponds with Fig 5B.
(MP4)

S5 Video Description: 3-Dimensional rendering of time-lapse confocal (AiryScan 4Y Multiplex) microscopy of PfCINCH-mNeonGreen; PfIMC1c-mCherry parasites. PfCINCH-mNeonGreen is visible in green, PfIMC1c is visible in magenta. Parasites were treated with a volume of DMSO equivalent to that required to bring the concentration of cytochalasin D to 2 μM in Video 6 for 4 hours before imaging; the same DMSO:media ratio was utilized in the imaging media. Z-stacks were collected every 20 minutes for 13 hours; frame rate is 0.5 seconds per time point. Scale bar = 1 μm. Corresponds with Fig 7A.
(MP4)

S6 Video Description: 3-Dimensional rendering of time-lapse confocal (AiryScan 4Y Multiplex) microscopy of PfCINCH-mNeonGreen; PfIMC1c-mCherry parasites. PfCINCH-mNeonGreen is visible in green, PfIMC1c is visible in magenta. Parasites were treated with 2 μM cytochalasin D for 4 hours before imaging and 2 μM cytochalasin D was utilized in the imaging media. Z-stacks were collected every 20 minutes for 13 hours; frame rate is 0.5 seconds per time point. Scale bar = 1 μm. Corresponds with Fig 7A.
(MP4)

S7 Video Description: 3-Dimensional rendering of time-lapse confocal (AiryScan 4Y Multiplex) microscopy of PfCINCH-mNeonGreen; PfIMC1c-mCherry parasites. PfCINCH-mNeonGreen is visible in green, PfIMC1c is visible in magenta. Parasites were treated with a volume of DMSO equivalent to that required to bring the concentration of jasplakinolide to 1 μM in Video 8 for 4 hours before imaging; the same DMSO:media ratio was utilized in the imaging media. Z-stacks were collected every 20 minutes for 13 hours; frame rate is 0.5 seconds per time point. Scale bar = 1 μm. Corresponds with Fig 7B.
(MP4)

S8 Video Description: 3-Dimensional rendering of time-lapse confocal (AiryScan 4Y Multiplex) microscopy of PfCINCH-mNeonGreen; PfIMC1c-mCherry parasites. PfCINCH-mNeonGreen is visible in green, PfIMC1c is visible in magenta. Parasites were treated with 1 μM jasplakinolide for 4 hours before imaging and 1 μM jasplakinolide was utilized in the imaging media. Z-stacks were collected every 20 minutes for 13 hours; frame rate is 0.5 seconds per time point. Scale bar = 1 μm. Corresponds with Fig 7B.
(MP4)

S9 Video Description: 3-Dimensional rendering of time-lapse confocal (AiryScan 4Y Multiplex) microscopy of PfCINCH-mNeonGreen; PfIMC1c-mCherry parasites. PfCINCH-mNeonGreen is visible in green, PfIMC1c is visible in magenta. Parasites were treated with 30 μM BAPTA-AM immediately before imaging and 30 μM BAPTA-AM was utilized in the imaging media. Z-stacks were collected every 20 minutes for 13 hours; frame rate is 0.5 seconds per time point. Scale bar = 1 μm. Corresponds with Fig 9A.
(MP4)

S10 Video Description: 3-Dimensional rendering of time-lapse confocal (AiryScan 4Y Multiplex) microscopy of PfCINCH-mNeonGreen; PfIMC1c-mCherry parasites. PfCINCH-mNeonGreen is visible in green, PfIMC1c is visible in magenta. Parasites were treated a volume of DMSO equivalent to that required to bring the concentration of BAPTA-AM to 30 μM in Video 9 immediately before imaging, and a proportionate volume of DMSO was added to the imaging media. Z-stacks were collected every 20 minutes for 13 hours; frame rate is 0.5 seconds per time point. Scale bar = 1 μm. Corresponds with Fig 9A.
(MP4)

S1 Data This file contains the primary data measurements for all figures. (XLSX)
