# Supplementary figures and images for: Elucidating the spatio-temporal dynamics of the Plasmodium falciparum basal complex
Source: PLoS Pathog. 2024 Jun 3;20(6):e1012265. doi: 10.1371/journal.ppat.1012265 (PMC11175456; doi:10.1371/journal.ppat.1012265)

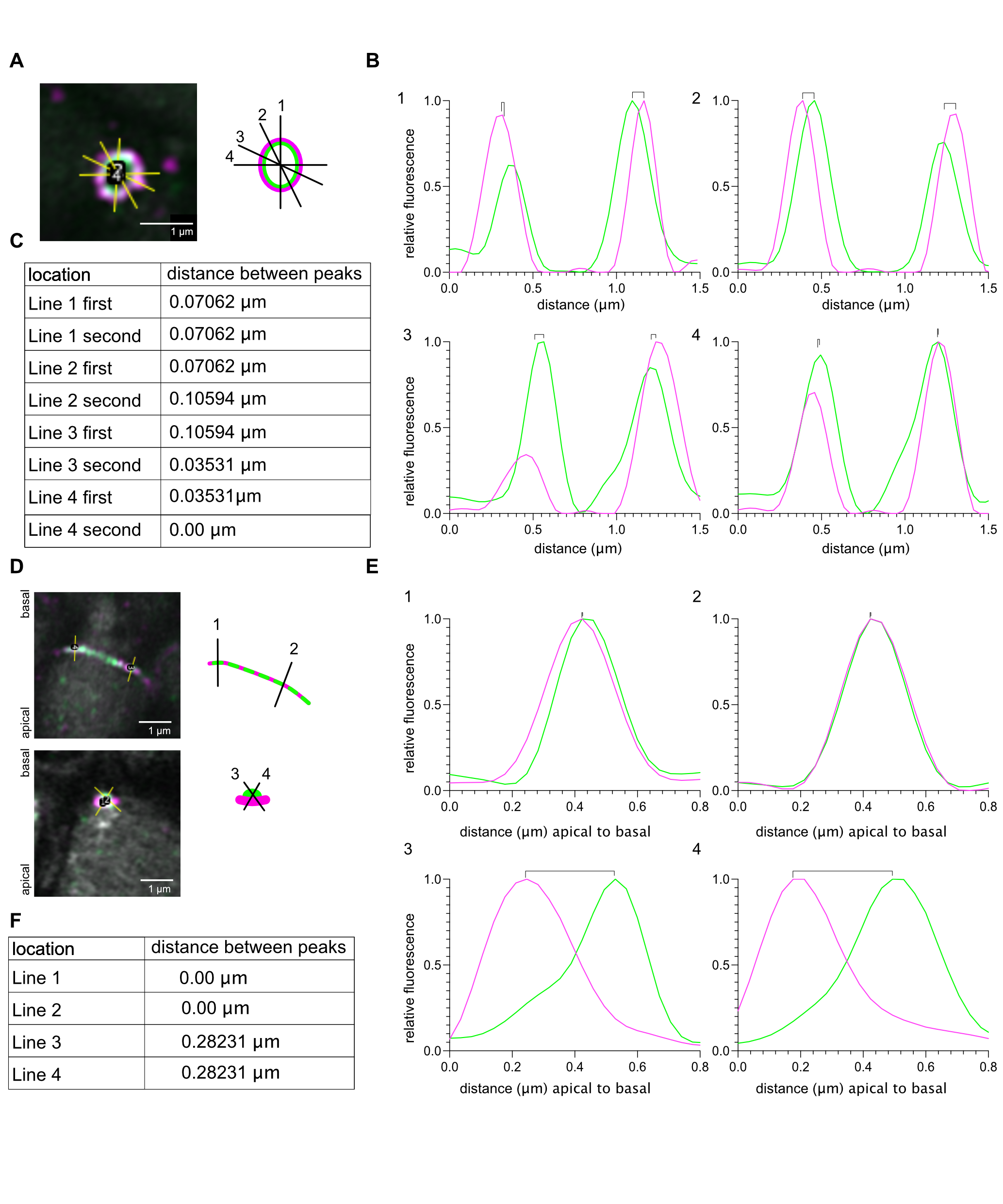

Supplement: S1 Fig — A) Example of PfMyoJ and PfCINCH dual tagged en face BC ring with lines drawn at 4 predetermined angles—U-ExM image (left) and diagram (right). B) Fluorescence intensity graphs overlaying PfMyoJ (green) and PfCINCH (magenta) intensity values, each corresponding with the relevant line in A). Black bars between the different colored peaks represent the distance between these two proteins by comparing the distance between their highest intensities. C) Calculated distance for each peak intersection measurement corresponding to the graphs in B). D) Example of PfMyoJ and PfCINCH dual tagged sideways BC ring with two perpendicular lines drawn across the ring from apical to basal in orientation. Top = mid segmentation U-ExM image (left) and model (right); Bottom = post-egress U-ExM image (left) and model (right). E) same as B) but for the lines drawn and labelled in D). F) same as C) but for distances demonstrated in D) and E). All scale bars = 1 μm. (TIFF) [file ppat.1012265.s001.tiff]

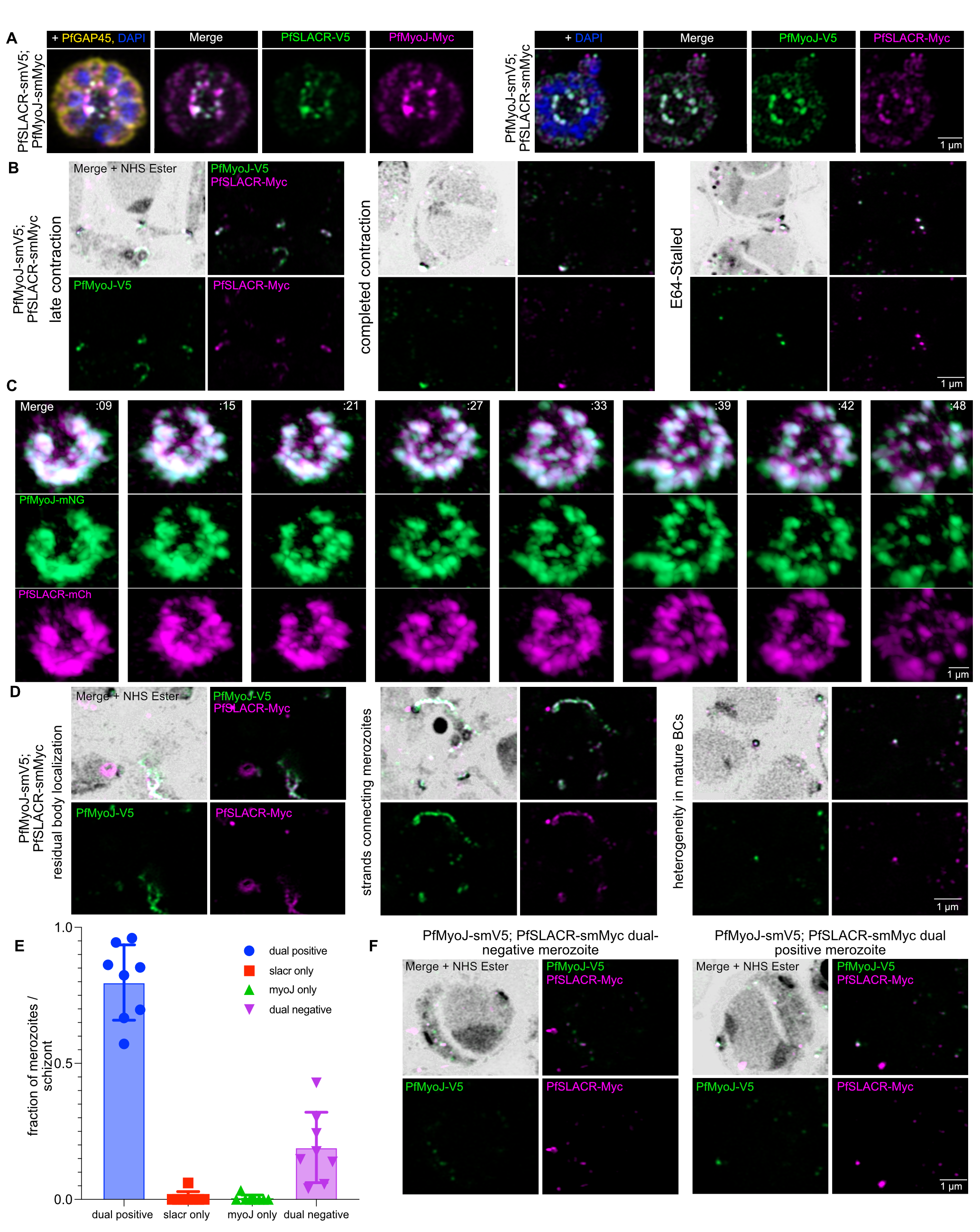

Supplement: S2 Fig — A) Immunofluorescence of two dual-tagged PfSLACR and PfMyoJ lines to show colocalization of proteins in extra-BC localizations, along with IMC protein PfGAP45. Left: PfSLACR-smV5, PfMyoJ-smMyc. Right: PfMyoJ-smV5; PfSLACR-smMyc. B) U-ExM slices of PfMyoJ-smV5; PfSLACR-smMyc parasites during late contraction, complete contraction, and post-egress (E64-stalled). C) Selected time points of live cell imaging of a PfMyoJ-mNeonGreen; PfSLACR-mCherry parasite. D) U-ExM slices of PfMyoJ-smV5; PfSLACR-smMyc parasites illustrating various non-basal complex localization phenotypes. E) Quantification of merozoite heterogeneity in post-egress PfMyoJ-smV5; PfSLACR-smMyc parasites. n = 8 schizonts with 25–37 merozoites/schizont per time point. F) Representation of dual positive and dual negative merozoite from the PfMyoJ-smV5; PfSLACR-smMyc parasite line. Both merozoites’ basal complexes were in the same focal plane. The data in E) are displayed as mean ± SD with individual values also presented. Time represented as hours:minutes. All scale bars = 1 μm. (TIFF) [file ppat.1012265.s002.tiff]

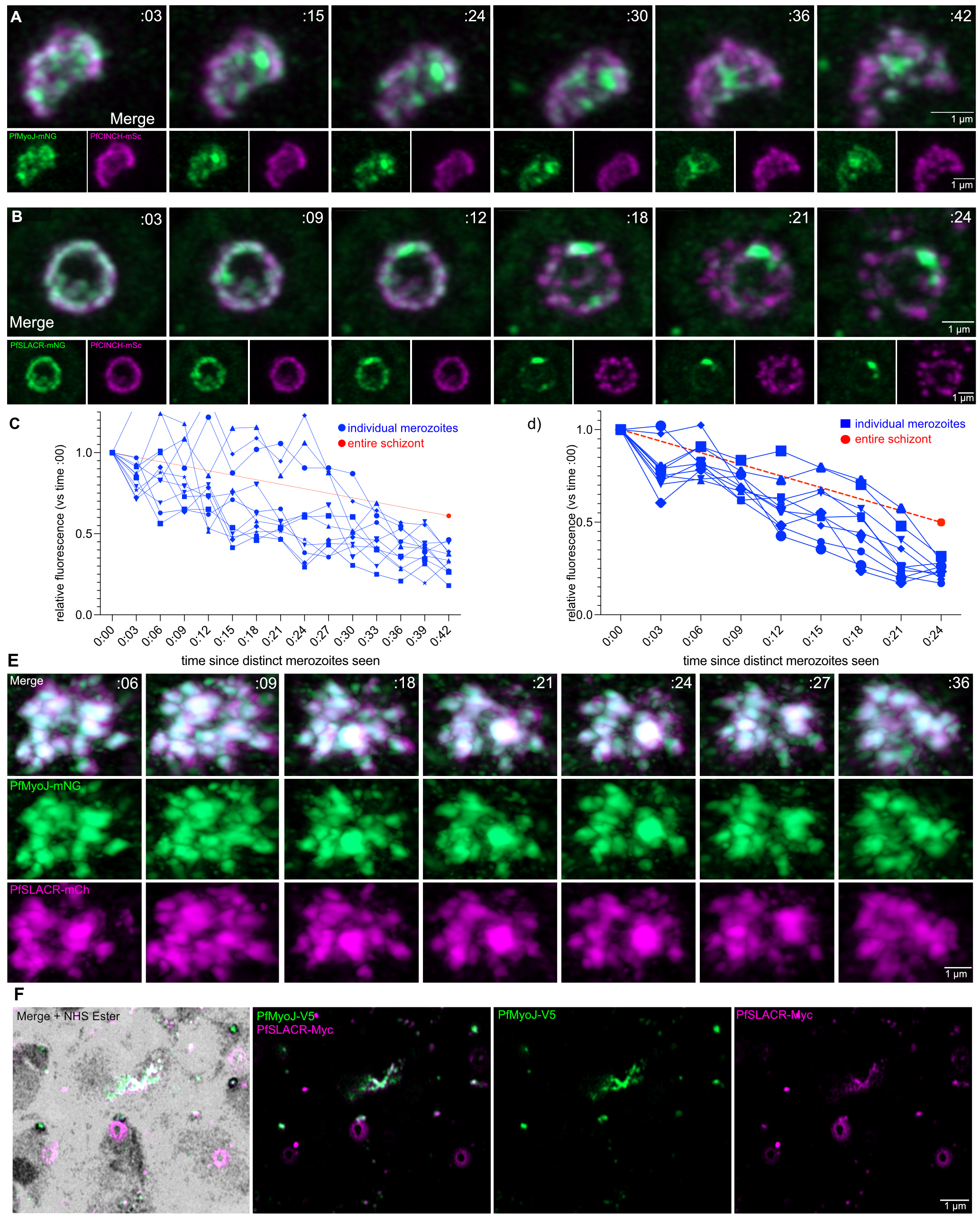

Supplement: S3 Fig — A) Selected time points of live cell imaging of PfMyoJ-mNeonGreen; PfCINCH-mScarlet parasite starting immediately pre-egress. B) Same as A) but with PfSLACR-mNeonGreen; PfCINCH-mScarlet parasite. C) Plot comparing changes in the fluorescence intensity of individual merozoites (in the PfMyoJ Channel) of the schizont represented in A) over time relative to changes in the entire schizont. D) Same as C), but with PfSLACR; graph made from data taken from schizont in B). E) Selected time points of live cell microscopy of PfMyoJ-mNeonGreen; PfSLACR-mCherry parasite starting immediately pre-egress. F) Additional U-ExM image of PfMyoJ-smV5; PfSLACR-smMyc parasite where PfMyoJ-smV5 and PfSLACR-smMyc colocalize in extra-merozoite protein agglomerates. All times represented as hours:minutes. All scale bars = 1 μm. (TIFF) [file ppat.1012265.s003.tiff]

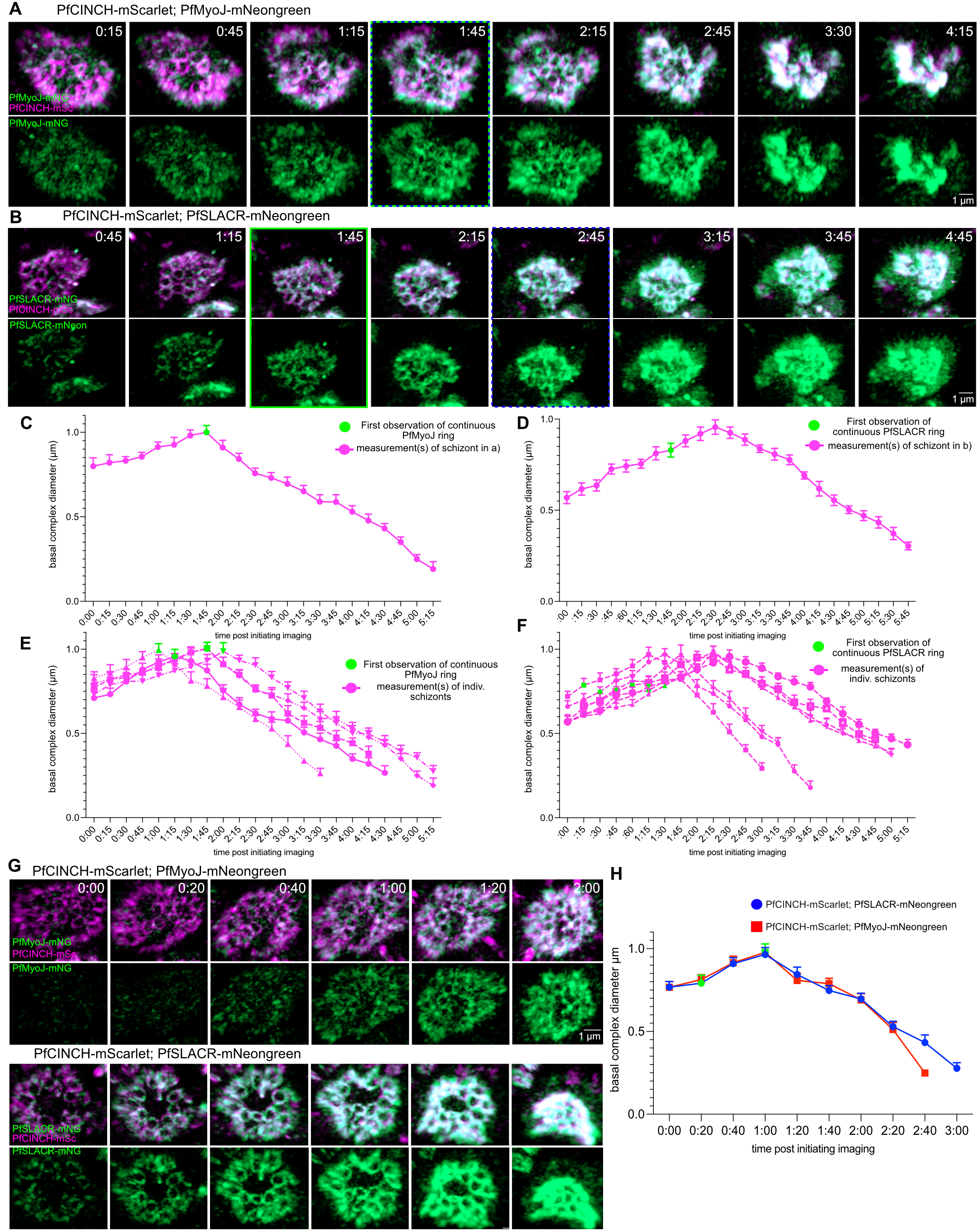

Supplement: S4 Fig — A) Selected time points of live cell live cell imaging of PfMyoJ-mNeonGreen; PfCINCH-mScarlet parasites. Green box = initial observation of contiguous PfMyoJ ring. Blue dashed box = time point when BC diameter is greatest. B) Same as A) but with PfSLACR-mNeonGreen; PfCINCH-mScarlet parasites. C) Graph of mean ring diameter of schizont in A) over time; pink line = measurements for schizont in A). Green point = initial observation of contiguous PfMyoJ ring. D) Graph of mean ring diameter of schizont in B) over time; pink line = measurements for schizont in B). Green point = initial observation of contiguous PfSLACR ring. E) Graph of mean ring diameter of multiple PfMyoJ-mNeonGreen; PfCINCH-mScarlet schizonts with clear, measurable rings over time; green points = initial observation of contiguous PfMyoJ ring in each parasite. F) Same, but with PfSLACR-mNeonGreen; PfCINCH-mScarlet schizonts. G) Selected time points from synchronized live cell microscopy experiment comparing BCD-matched PfMyoJ-mNeongreen; PfCINCH-mScarlet parasites (top two rows) and PfSLACR-mNeonGreen; PfCINCH-mScarlet parasites (bottom two rows). H) graph of mean BCD for parasites in G) over time, with the green point representing initial observation of contiguous ring of each protein. Data in C), D), E), F), H) represented as mean ± SD. Time represented as hours:minutes. All scale bars = 1 μm. (TIFF) [file ppat.1012265.s004.tiff]

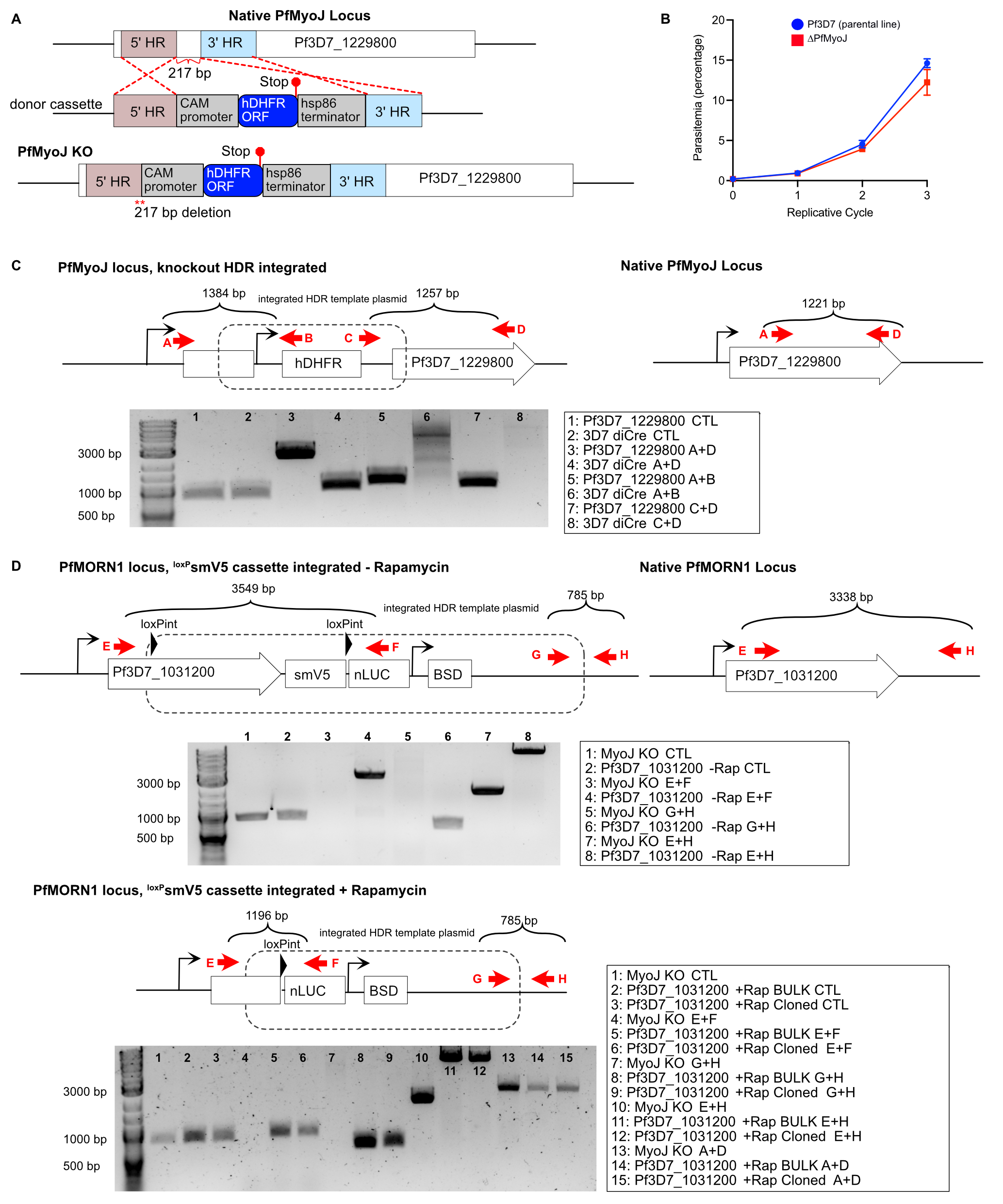

Supplement: S5 Fig — A) Diagram demonstrating ΔPfMyoJ creation strategy using double-crossover mediated integration and inducing a 217-base pair deletion between the HRs as well as the insertion of the donor cassette. B) Second replicate of flow cytometry-based replication curve comparing ΔPfMyoJ parasites to parental 3D7-DiCre line. C) Diagram comparing the PfMyoJ genomic locus upon integration of the disrupting HDR plasmid (dotted box) to the native genomic locus and integration PCRs for ΔPfMyoJ, confirming the disruption of this locus, with primers A & B, C & D, A & D or CTL primers (oJDD5078/5079 in. S1 Table). D) Diagram comparing the PfMORN1 genomic locus upon integration of the HDR plasmid (dotted box) to the native genomic locus as well as the integrated locus upon addition of rapamycin. Integration PCRs for PfMORNloxP-smV5, demonstrating successful excision of the majority of PfMORN1 in the bulk population as well as cloning of an excised dual-knockout parasite with primers E & F, G & H, E & H or CTL primers (oJDD5078/5079 in S1 Table). (TIFF) [file ppat.1012265.s005.tiff]

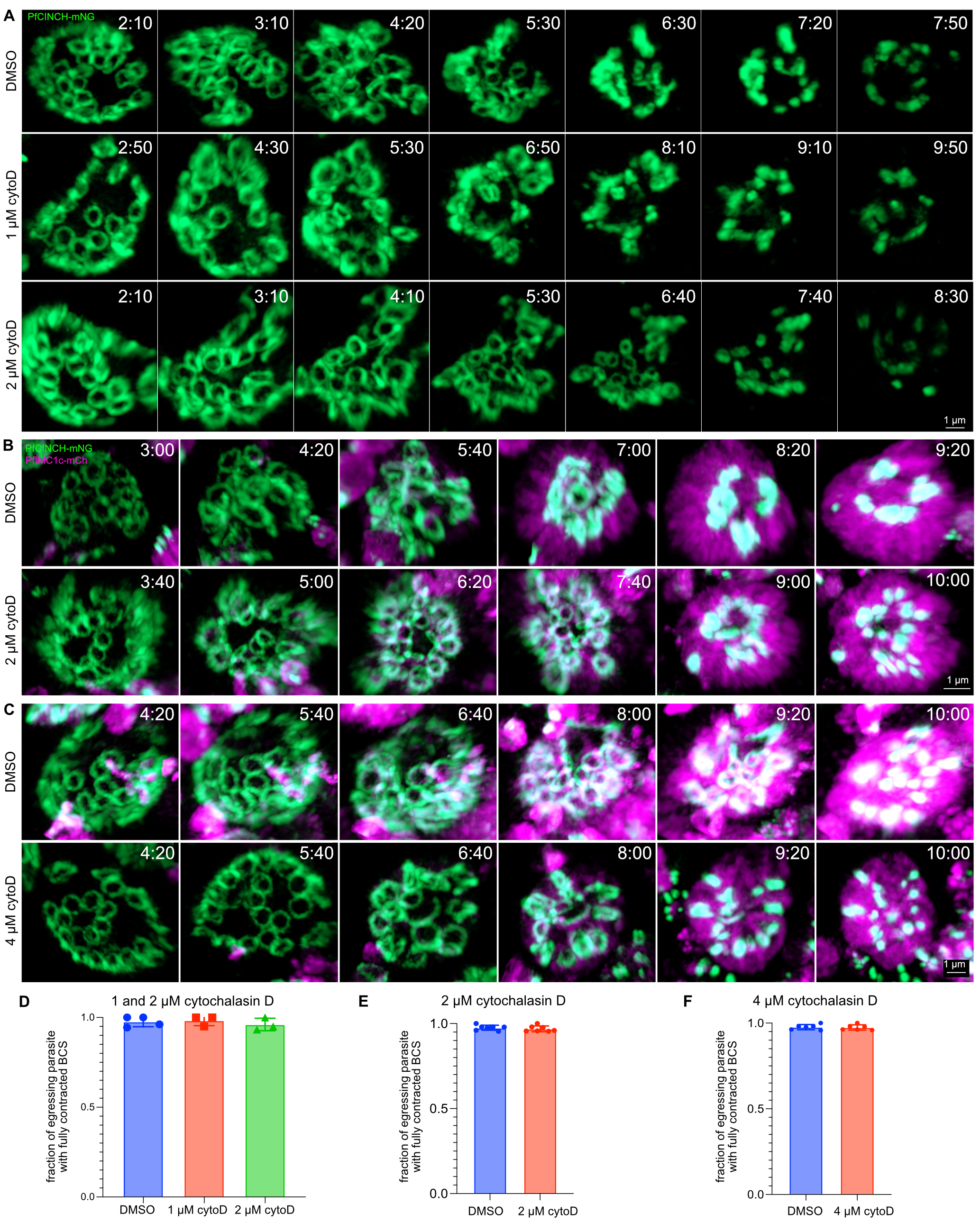

Supplement: S6 Fig — A) Selected time points of live cell imaging showing basal complex contraction in PfCINCH-mNeonGreen, PfIMC1c-mCherry parasites treated with DMSO (top row) 1 μM (middle row) or 2 μM (bottom row) cytoD. B) Selected time points of live cell imaging showing basal complex contraction in PfCINCH-mNeonGreen; PfIMC1c-mCherry parasites treated with DMSO (top row) or 2 μM (bottom row) cytoD. C) Same as b but with 4 μM cytoD. D) Comparison of fraction of egressing parasites per field with fully contracted basal complexes (ie, BCs that contracted to the point that their diameter could not be measured) between DMSO, 1 μM, and 2 μM cytoD treated parasites depicted in A). n = 3–4 fields/condition, 20–30 parasites/field. E) Comparison of fraction of egressing parasites per field with fully contracted basal complexes between DMSO and 2 μM cytoD parasites depicted in B). n = 6 fields/condition, 30–50 parasites/field. F) Comparison of fraction of egressing parasites per field with fully contracted basal complexes between DMSO and 4 μM cytoD parasites depicted in C). n = 6 fields/condition, 30–50 parasites/field. Time represented as hours: minutes. For D)-F), data are displayed as mean ± SD with individual values overlayed. All scale bars = 1 μm. (TIFF) [file ppat.1012265.s006.tiff]

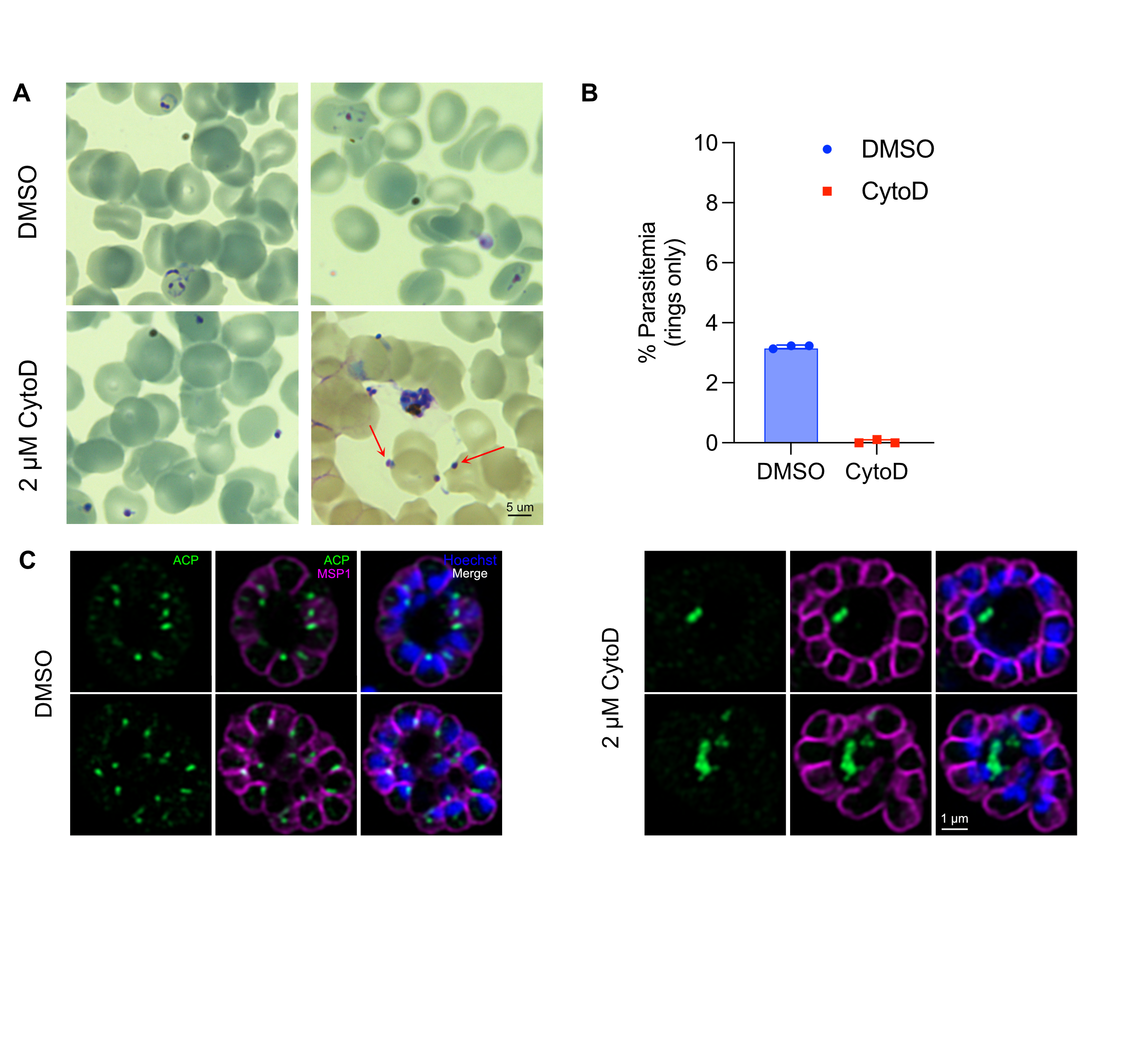

Supplement: S7 Fig — A) Field’s stain images of resulting parasitemia taken 4 hours after percoll-purified schizonts were added to red blood cells with 2 μM cytochalasin D or an equivalent volume of DMSO were added. B) Quantification of ring parasitemia in wells imaged in A). The data are displayed as mean ± SD with individual values overlayed; reinvasion assays were performed in triplicate and these data represent one of two biological replicates. C) Immunofluorescence of late schizonts treated with DMSO or 2 μM cytochalasin D comparing distribution of plasma membrane protein PfMSP1 and apicoplast protein PfACP. (TIFF) [file ppat.1012265.s007.tiff]

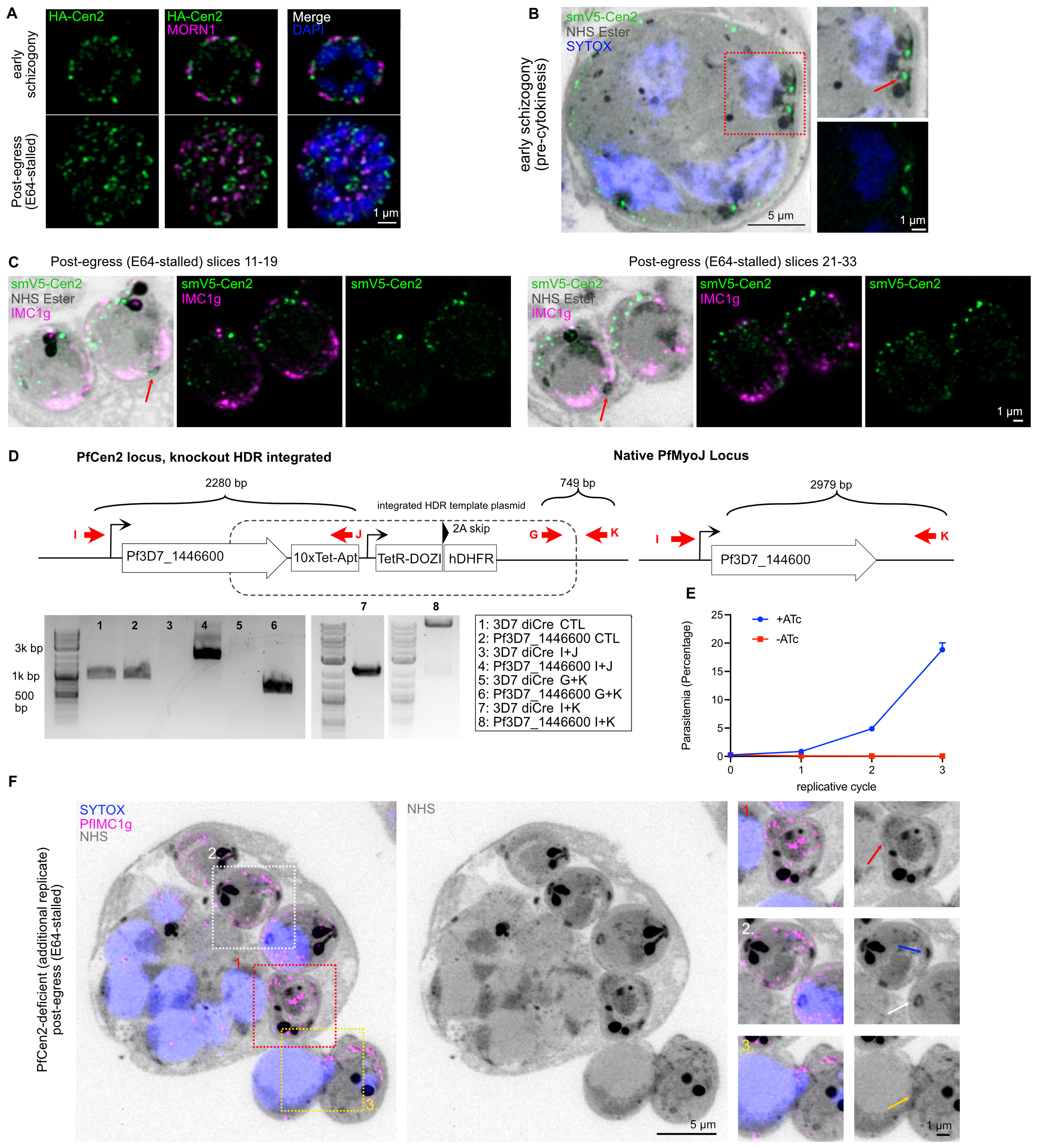

Supplement: S8 Fig — A) Immunofluorescence of episomally-expressed 2HA-PfCen2 in early schizogony, pre-cytokinesis, and post-egress (E64-stalled) with basal complex marker PfMORN1. B) U-ExM of episomally-expressed smV5-Cen2 and far-red DNA dye SYTOX in early schizogony, pre-cytokinesis, demonstrating localization of smV5-PfCen2 to the outer centriolar plaque. Red box indicates the 1.5x-zoomed region to the right of this panel, in which a red arrow points to smV5-PfCen2 localization in the centriolar plaque. C) U-ExM of episomally-expressed smV5-Cen2 and IMC-associated alveolin protein PfIMC1g in post-egress merozoites (from E64-stalled culture). Two different projections of 8–10 slices are shown; red arrows point to localization of smV5-PfCen2 to the basal cup in these mature merozoites. D) Diagram comparing the PfCen2 genomic locus upon integration of the disrupting HDR plasmid (dotted box) to the native genomic locus and integration PCRs for PfCen2Tet, confirming the disruption of this locus, with primers I & J, G & K, I & K or CTL primers (oJDD5078/5079 in S1 Table). E) Second replicate of flow cytometry-based replication curve comparing PfCen2Tet parasites in the presence (PfCen2-sufficient) and absence (PfCen2-deficient) of ATc. F) U-ExM slices of additional post-egress (E64 stalled) PfCen2-deficient PfCen2Tet parasite. Numbered, dashed boxes indicate the respective numbered 1.5x-zoomed region to the right of the panels; red, white, and yellow arrows point to enlarged basal complexes of various sizes and blue arrow indicates a normally sized basal complex. All scale bars = 1 μm except the non-zoomed U-ExM panels in F) where scale bar = 5 μm. (TIFF) [file ppat.1012265.s008.tiff]

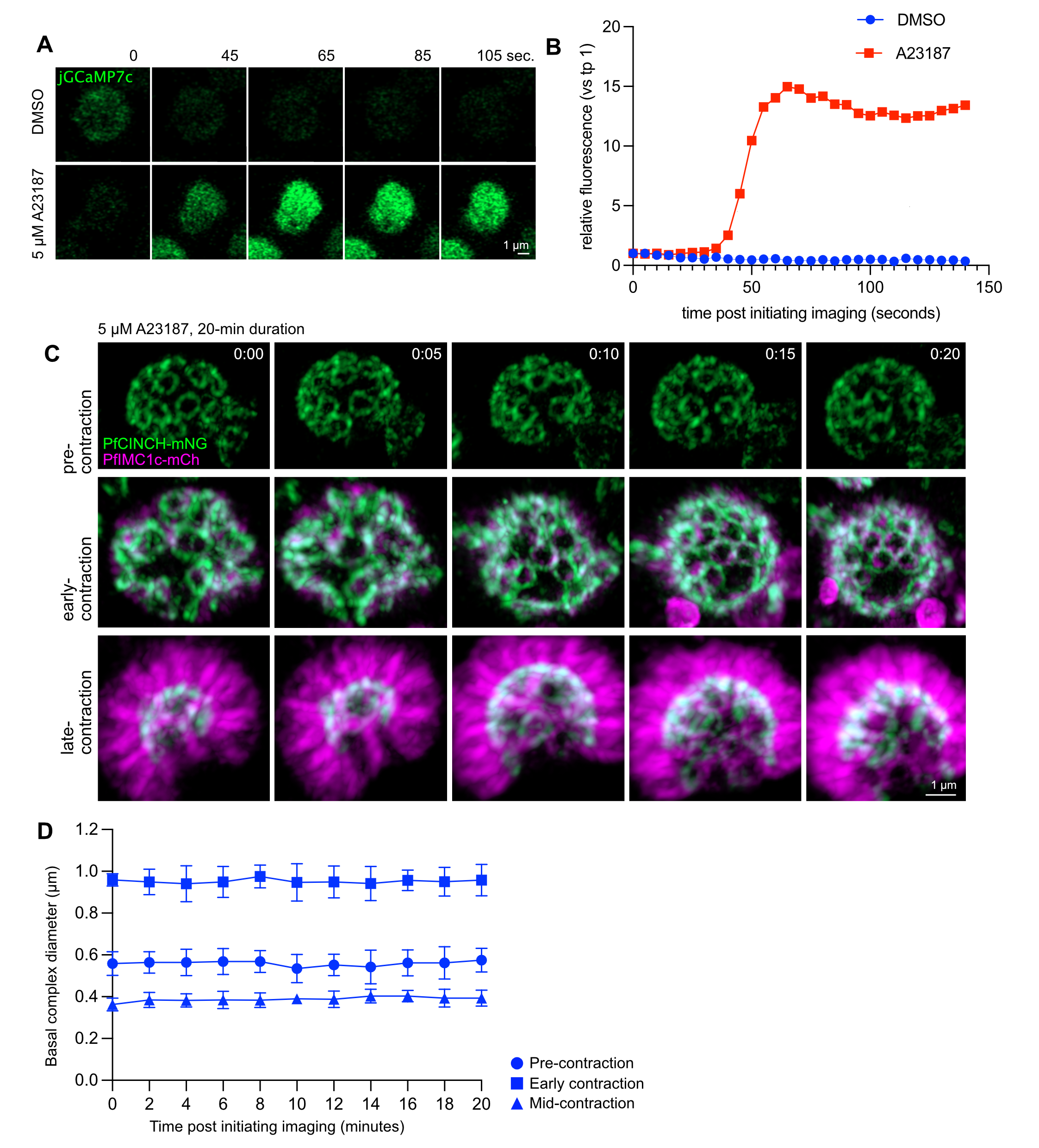

Supplement: S9 Fig — A) Selected time points of live cell imaging showing a single slice of jGCaMP7c-expressing parasites after the addition of either DMSO or 5 μM A23187, validating the efficacy of this compound. Time represented as seconds post initiating imaging. B) Quantification of A); mean fluorescence intensity of the parasite depicted in A) was calculated for each image taken at each 5-second interval and normalized to fluorescence intensity of the first image, immediately after initiating imaging. C) Selected time points of live cell imaging of PfCINCH-mNG; PfIMC1c-mCherry (mCh) parasites starting at different stages of schizogony: pre-contraction (top row), early-contraction (middle row), and late-contraction (bottom row) after the addition of 5 μM A23187, imaged for 20 minutes with 2.5 minute intervals. Time represented as hours:minutes. D) Graph of measured BCD for parasites in C) over the course of imaging. Stage of segmentation is matched by symbol. All scale bars = 1 μm. (TIFF) [file ppat.1012265.s009.tiff]
